# Systemic ceramide accumulation leads to severe and varied pathological consequences

**DOI:** 10.1002/emmm.201202301

**Published:** 2013-05-16

**Authors:** Abdulfatah M Alayoubi, James C M Wang, Bryan C Y Au, Stéphane Carpentier, Virginie Garcia, Shaalee Dworski, Samah El-Ghamrasni, Kevin N Kirouac, Mathilde J Exertier, Zi Jian Xiong, Gilbert G Privé, Calogera M Simonaro, Josefina Casas, Gemma Fabrias, Edward H Schuchman, Patricia V Turner, Razqallah Hakem, Thierry Levade, Jeffrey A Medin

**Affiliations:** 1 Institute of Medical Science, University of TorontoToronto, Ontario, Canada; 2 College of Medicine, Taibah UniversityMadinah, Saudi Arabia; 3 University Health NetworkToronto, Ontario, Canada; 4 INSERM UMR1037, Centre de Recherches en Cancérologie de Toulouse, Université Toulouse III Paul-SabatierToulouse, France; 5 Department of Biochemistry, University of TorontoToronto, Ontario, Canada; 6 Department of Medical Biophysics, University of TorontoToronto, Ontario, Canada; 7 Department of Genetics and Genomic Sciences, Mount Sinai School of MedicineNew York, NY, USA; 8 Research Unit on Bioactive Molecules, Department of Biomedicinal Chemistry, Institute for Advanced Chemistry of Catalonia, Spanish National Research CouncilBarcelona, Spain; 9 Department of Pathobiology, University of GuelphGuelph, Ontario, Canada; 10 Laboratoire de Biochimie Métabolique, Institut Fédératif de BiologieCHU de Toulouse, Toulouse, France

**Keywords:** acid ceramidase, Lysosomal storage disorders, Farber disease, MCP-1

## Abstract

Farber disease (FD) is a severe inherited disorder of lipid metabolism characterized by deficient lysosomal acid ceramidase (ACDase) activity, resulting in ceramide accumulation. Ceramide and metabolites have roles in cell apoptosis and proliferation. We introduced a single-nucleotide mutation identified in human FD patients into the murine *Asah1* gene to generate the first model of systemic ACDase deficiency. Homozygous *Asah1*^P361R/P361R^ animals showed ACDase defects, accumulated ceramide, demonstrated FD manifestations and died within 7–13 weeks. Mechanistically, MCP-1 levels were increased and tissues were replete with lipid-laden macrophages. Treatment of neonates with a single injection of human ACDase-encoding lentivector diminished the severity of the disease as highlighted by enhanced growth, decreased ceramide, lessened cellular infiltrations and increased lifespans. This model of ACDase deficiency offers insights into the pathophysiology of FD and the roles of ACDase, ceramide and related sphingolipids in cell signaling and growth, as well as facilitates the development of therapy.

→See accompanying article http://dx.doi.org/10.1002/emmm.201302781

## INTRODUCTION

Farber disease (FD) is a rare and severe autosomal recessive lysosomal storage disorder (LSD) (Levade et al, 2009). It is characterized by a deficiency of acid ceramidase (ACDase) activity (Sugita et al, 1972) that results in the intralysosomal accumulation of ceramide in various tissues. The disorder is manifested early after birth as voice hoarseness, arthritis, and subcutaneous nodules (Levade et al, 1995). Neurological and visceral involvement are also observed (Antonarakis et al, 1984; Pellissier et al, 1986) that lead to psychomotor retardation and hepatosplenomegaly, respectively. Most FD patients show growth and developmental impairment and do not live past 2 years of age (Haraoka et al, 1997). The lack of specific treatment for FD has limited management of the disorder primarily to supportive care. While bone marrow transplantation has been reported to be effective in relieving joint contractures and subcutaneous nodules (Yeager et al, 2000), neurologic symptoms and patient longevity were found to be largely unaffected.

Several mutations in the acid ceramidase gene (*ASAH1*) cause low ACDase activities in Farber patients (Bar et al, 2001). Yet the mechanisms by which ACDase deficiency leads to the development of the Farber phenotype remain essentially unknown (Zhang et al, 2000). Widespread infiltration of lipid-laden macrophages into various organs is thought to cause some symptoms observed in Farber patients. However, the link between the build-up of ceramide primarily in lysosomes and infiltration of macrophages is still unclear. Ceramide may also exhibit its deleterious effects through its signaling properties (Burek et al, 2001). In this regard, ACDase is a key regulator of the balance between the pro-apoptotic substrate ceramide and the mitogenic metabolite sphingosine-1-phosphate (Lucki and Sewer, 2011). Importantly, a previous attempt to completely “knock-out” the murine *Asah1* gene resulted in early embryonic lethality (Li et al, 2002) and the reduction of ACDase in mouse ovaries in a conditional “knock-out” model led to apoptosis of oocytes (Eliyahu et al, 2012), underscoring the broad importance of this pathway.

Here we report the generation of the first viable model of systemic ceramide accumulation via “knock-in” of a known human *ASAH1* mutation [Proline (P) 362 to Arginine (R)] into a conserved murine *Asah1* gene locus (P361R). We also present new mechanistic insights that help explain the progression of this severe disorder. Lastly, we show as proof-of-principle in this model that neonatal gene therapy using recombinant lentivectors (LVs) encoding human ACDase is a feasible therapeutic platform for the treatment of FD.

## RESULTS

### Mouse embryonic fibroblasts (MEFs) from *Asah1*^P361R/P361R^ mice are deficient in ACDase activity

Gene targeting was used to generate *Asah1*^P361R^ knock-in embryonic stem (ES) cells and mice (Supporting Information Fig S1). Inter-crossings of heterozygous mice yielded offspring *Asah1*^P361R/P361R^ mice in the expected Mendelian ratio (Supporting Information Table S1). MEFs isolated and grown from wild-type (WT), heterozygous, and *Asah1*^P361R/P361R^ embryos were loaded with [^3^H-ceramide]-labeled sphingomyelin for 40 h. Lipids were then extracted and quantified. Deficient ACDase activity in cells from *Asah1*^P361R/P361R^ animals was evident by accumulation of lysosomal ceramide. Whereas undegraded ceramide represented 22% of sphingomyelin metabolites in WT MEFs and 30% in heterozygous MEFs, it accounted for 62 and 68% in two distinct MEF cell lines from *Asah1*^P361R/P361R^ embryos (data not shown). In a repeat independent experiment, undegraded ceramide represented 81 and 86% in two distinct MEF cell lines from *Asah1*^P361R/P361R^ embryos as compared to only 10% in WT MEFs (data not shown). Of note, degradation of sphingomyelin in MEFs from *Asah1*^P361R/P361R^ embryos was normal.

### Homozygous *Asah1*^P361R/P361R^ mice exhibit characteristic signs of Farber disease

**Figure 1:**
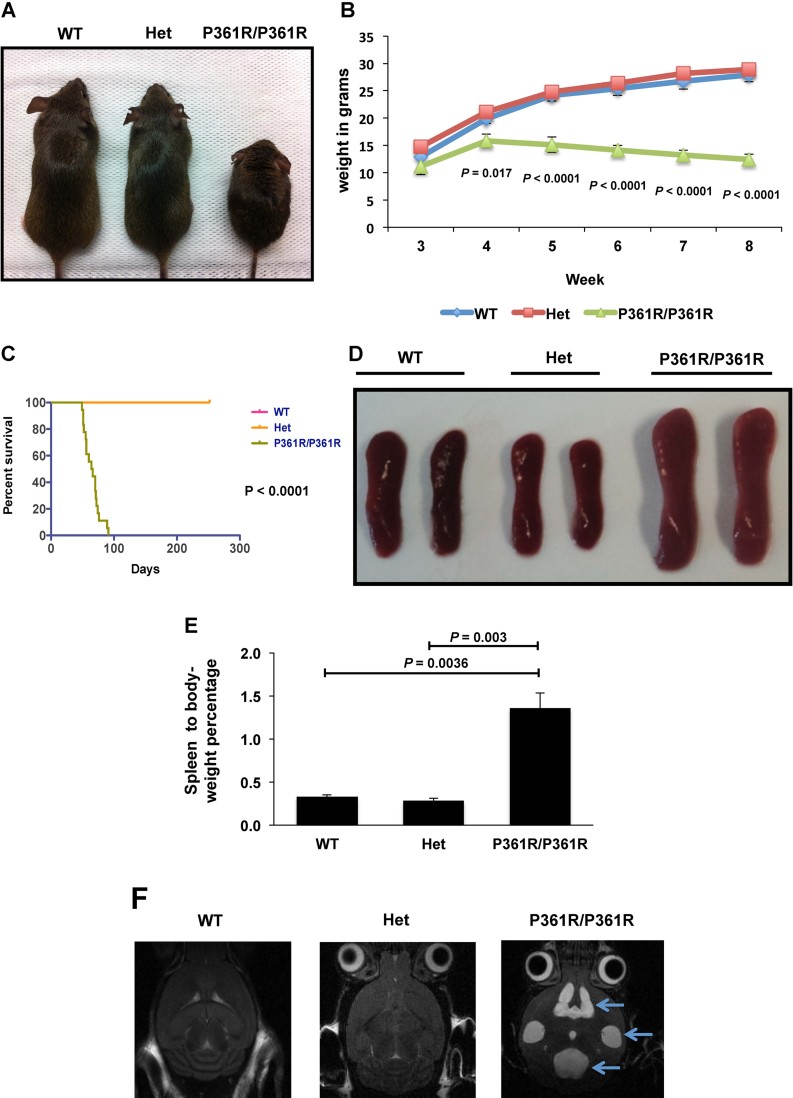
Growth defects, splenomegaly, and premature death of *Asah1*^P361R/P361R^ mice. General assessment of *Asah1*^P361R/P361R^ mice A photograph of 10-week-old wild-type (WT), heterozygous (Het), and *Asah1*^P361R/P361R^ mice.Growth curves measured in weights *versus* age (*n* = 10 of each genotype). Error bars represent standard errors of the mean.Kaplan–Meier survival analysis (*n* = 16 for each genotype).A photograph of spleens from each genotype.Ratios of spleen weights to body weights (*n* = 5 for heterozygous (Het) and *Asah1*^P361R/P361R^ mice; *n* = 3 for WT). Bars represent mean values. Error bars represent standard errors of the mean.Brain MRI scans of 10-week-old WT, heterozygous, and *Asah1*^P361R/P361R^ littermates. Hydrocephaly was detected in 5 out of 7 *Asah1*^P361R/P361R^ mice. Arrows indicate dilated brain ventricles.

**Figure 2:**
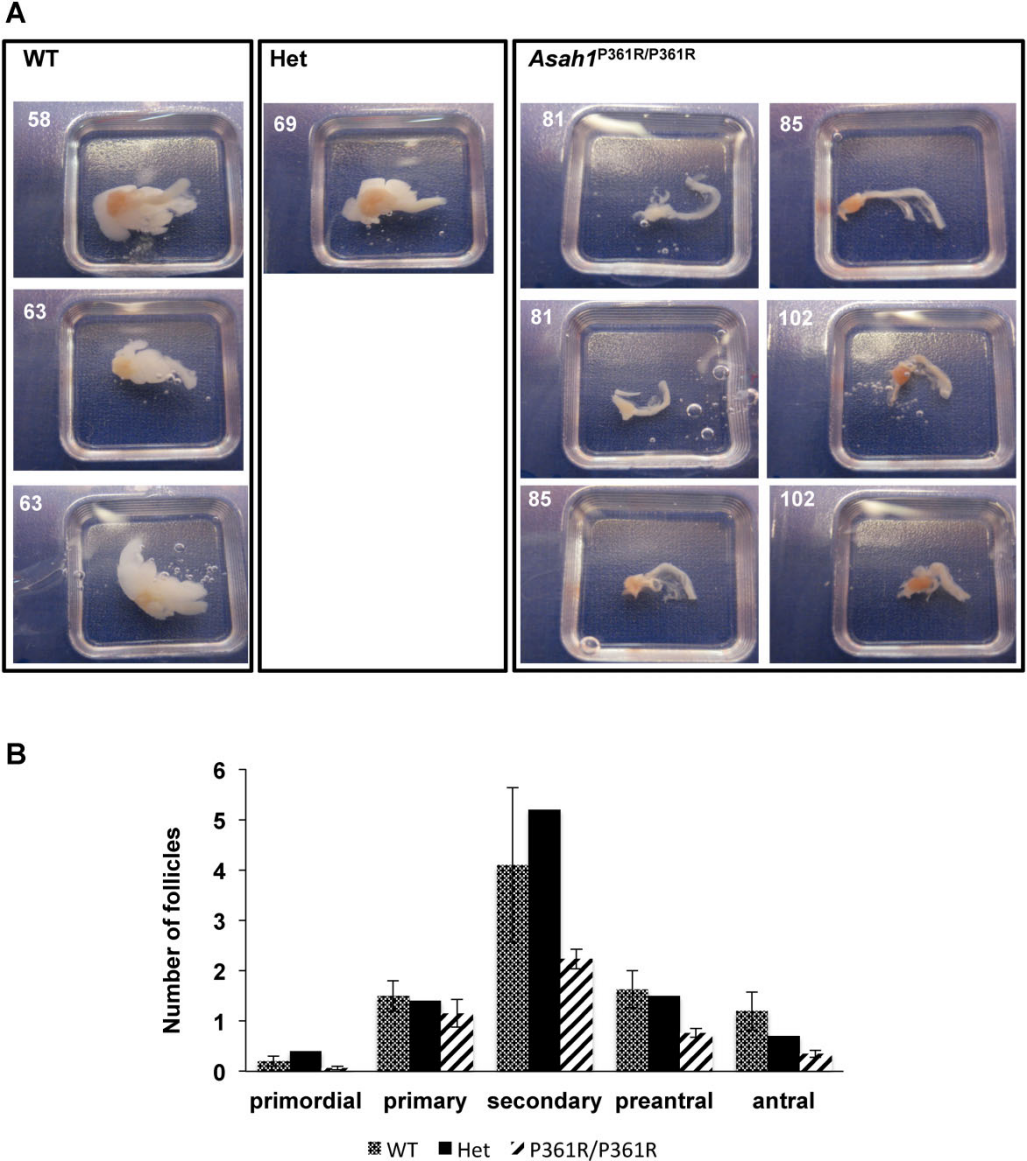
Impaired development of the ovaries in *Asah1*^P361R/P361R^ mice Pictures of ovaries from 9-week-old WT, heterozygous, and *Asah1*^P361R/P361R^ mice showing size differences.Analysis of follicular numbers at different stages in 9-week-old WT (*n* = 2), heterozygous (*n* = 1), and *Asah1*^P361R/P361R^ mice (*n* = 3). This analysis of follicular numbers was done once. Bars represent mean values. Error bars represent standard errors of the mean.

We observed that *Asah1*^P361R/P361R^ mice started to manifest growth retardation in comparison to their heterozygous and WT littermates as early as 3 weeks of age (Fig 1A and B). Indeed, *Asah1*^P361R/P361R^ mice reached maximum weight at 4 weeks. Afterwards, *Asah1*^P361R/P361R^ mice showed continuous and progressive weight loss (Fig 1B) punctuated by eventual death at approximately 7–13 weeks (Fig 1C). *Asah1*^P361R/P361R^ mice manifested lethargy, general dystrophy, and a weak forelimb grasp, which worsened with advancing age. Penile prolapse was also observed in male *Asah1*^P361R/P361R^ mice. In contrast, heterozygotes appeared phenotypically normal, showed growth patterns similar to their WT littermates (Fig 1B), and were asymptomatic over one year of age. Gross examination of viscera revealed remarkably enlarged spleens, thymuses, and lymph nodes (axillary, cervical, and inguinal) in *Asah1*^P361R/P361R^ mice. Spleens were firm and pale (Fig 1D) and the relative spleen:body-weight ratios were significantly increased (Fig 1E). Total-body MRI scans of 10 week-old *Asah1*^P361R/P361R^ mice (*n* = 7) showed massive hydrocephaly in five animals (Fig 1F). Scans from WT (*n* = 5) and heterozygous (*n* = 2) animals were unremarkable (Fig 1F). Ovaries from *Asah1*^P361R/P361R^ mice were smaller in size and covered with less fat compared to ovaries from WT animals (Fig 2A). Microscopic analysis of the ovaries showed a trend to lower numbers of follicles – especially at the antral stage – in *Asah1*^P361R/P361R^ mice (Fig 2B**)**.

### *Asah1*^P361R/P361R^ mice exhibit irregular peripheral blood features

**Figure 3:**
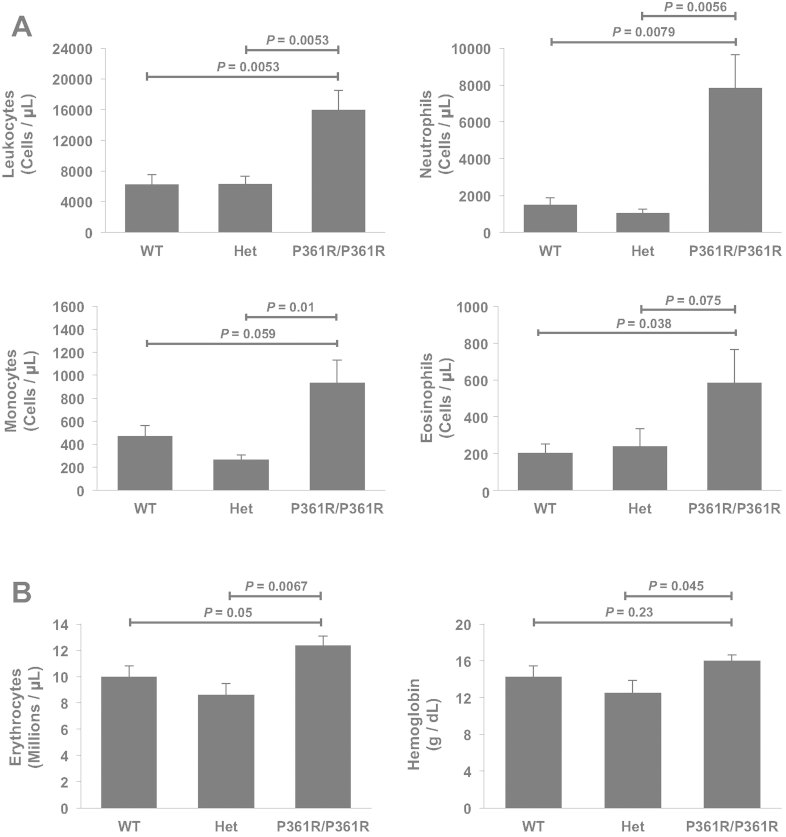
Altered hematopoiesis in *Asah1*^P361R/P361R^ mice **A,B.** Blood samples were collected from all animals at 7–10 weeks. Total leukocyte, monocyte, neutrophil, and eosinophil counts were determined (A); and (B) erythrocytes and hemoglobin levels were measured in WT (*n* = 10), heterozygous (*n* = 7), and *Asah1*^P361R/P361R^ mice (*n* = 9). The cell counts were measured once. Bars represent mean values. Error bars represent standard errors of the mean.

Leukocytes play a role in the pathogenesis of FD (Qualman et al, 1987). Total white blood cell counts were dramatically elevated in 7- to 10-week-old *Asah1*^P361R/P361R^ animals, while cell counts in heterozygotes were the same as those seen in WT animals (Fig 3A). Significantly higher levels of neutrophils, monocytes, and eosinophils were also observed in *Asah1*^P361R/P361R^ mice compared to control littermates (Fig 3A). Lymphocytes and basophils did not show significant differences although a trend to higher levels in *Asah1*^P361R/P361R^ mice was observed (data not shown). Red blood cells and hemoglobin levels were significantly increased above heterozygous controls (Fig 3B).

### *Asah1*^P361R/P361R^ mice accumulate high levels of ceramide and demonstrate reduced ACDase activities in lysates from various organs

**Figure 4:**
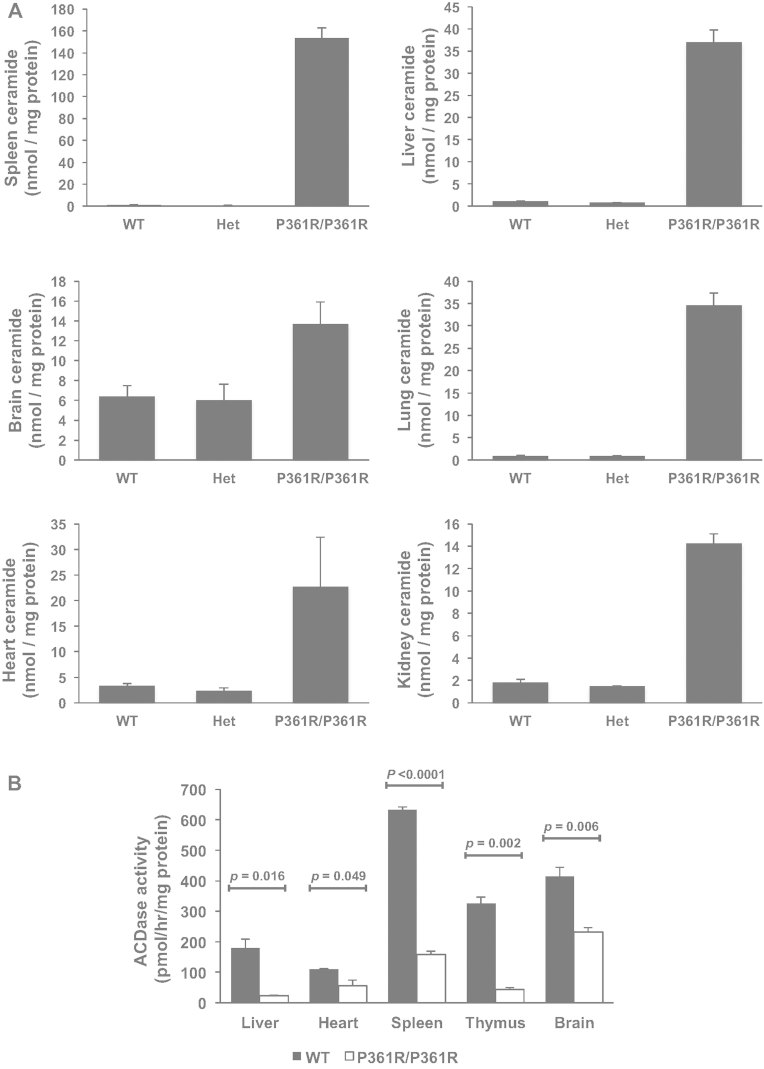
Accumulation of ceramide and reduction of ACDase activity in various tissues of *Asah1*^P361R/P361R^ mice Samples from spleens, livers, brains, lungs, hearts, and kidneys of 7- to 10-week-old mice were analyzed for total ceramide using an *E. coli* DAG kinase assay (*n* = 2 for all genotypes). The data points represent the average values from two independent experiments. Bars represent mean values. Error bars represent standard errors of the mean.Samples from livers, hearts, spleens, thymuses, and brains of 7- to 10-week-old mice were analyzed for ACDase activity by a liposome-based TLC assay using C12-NBD-Ceramide as the substrate at pH 4.6 (*n* = 3). This experiment was done once. Bars represent mean values. Error bars represent standard errors of the mean.

Total ceramide levels in various organs of *Asah1*^P361R/P361R^ mice and controls at 8 weeks of age were quantified. In all tested organs (spleen, brain, heart, liver, lungs, and kidneys), total ceramide levels were dramatically elevated in *Asah1*^P361R/P361R^ mice compared to WT mice; heterozygous animals displayed no increases in ceramide accumulation (Fig 4A). Similar results were obtained when ceramide levels were measured using internally-controlled ratios of ceramide to diacylglycerol (DAG; Supporting Information Fig S2). Importantly, ACDase protein expression levels in tissues from *Asah1*^P361R/P361R^ mice were similar to that from heterozygous and WT mice (Supporting Information Fig S3).

ACDase activities in lysates from various organs from *Asah1*^P361R/P361R^ and WT animals were evaluated at 7–10 weeks by a liposome-based assay. In lysates from all tested organs (liver, heart, spleen, thymus, and brains), ACDase activities were significantly reduced in *Asah1*^P361R/P361R^ mice compared to those derived from WT animals (Fig 4B). Spleens, thymuses, and livers showed the most severe reductions of ACDase activities in *Asah1*^P361R/P361R^ animals.

### Histiocytic infiltrations and characteristic Farber bodies are manifested in tissues of *Asah1*^P361R/P361R^ mice

**Figure 5:**
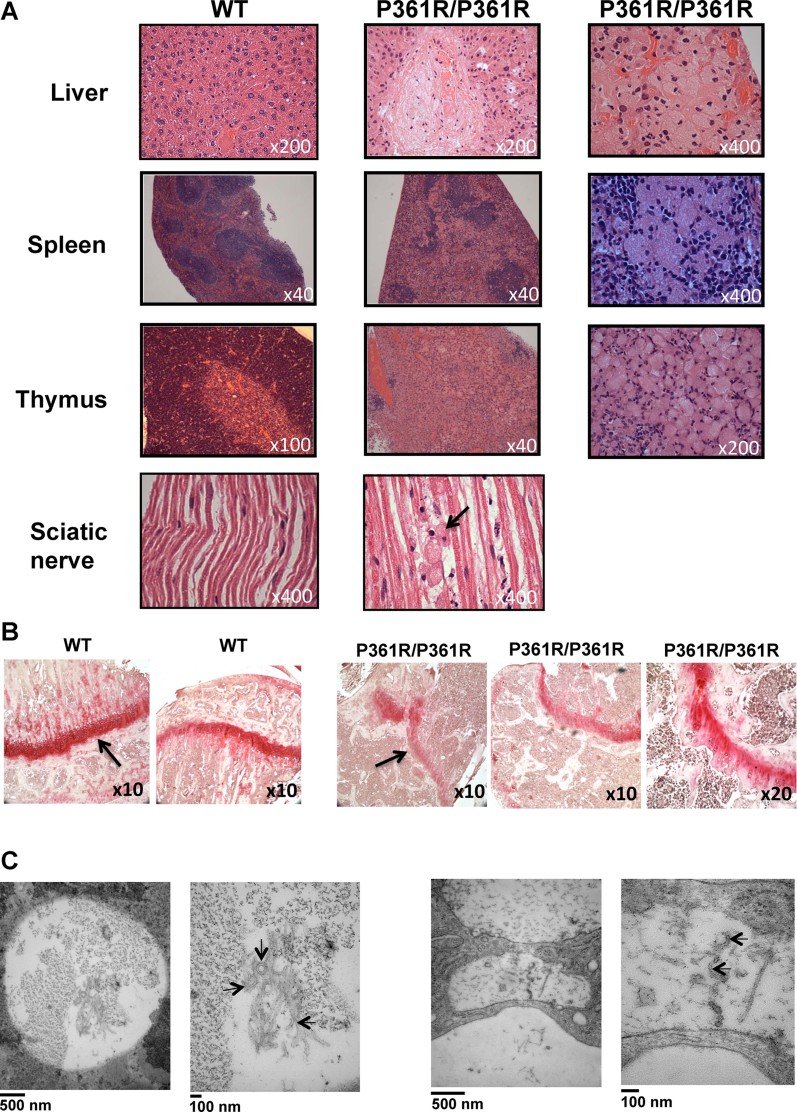
Infiltrating macrophages and pathognomonic Farber bodies in *Asah1*^P361R/P361R^ mice H&E staining of liver, spleen, thymus, and sciatic nerve of 7- to 10-week-old mice (arrow indicates infiltrating macrophages).Safranin-O staining of femurs from 7-week-old mice shows thinner epiphyseal growth plates in *Asah1*^P361R/P361R^ mice compared to a WT littermate (arrows).Electron microscopy images of hepatic (left panel) and peripheral nerve (right panel) sections from *Asah1*^P361R/P361R^ mice. Arrows indicate Farber bodies.

Histopathologic examination of H&E-stained tissues indicated moderate-to-marked histiocytic infiltrates within the liver, spleen, and thymus of 7- to 10-week-old *Asah1*^P361R/P361R^ animals with expansion and disruption of the parenchyma (Fig 5A). Infiltrating histiocytes had abundant pale, foamy-to-finely granular, eosinophilic cytoplasms with displaced nuclei. Histiocytic infiltrates were accompanied by mild-to-moderate neutrophilic infiltrates. Patchy single-cell necrosis to random, multifocal areas of acute hepatocellular necrosis were also noted. Similar histiocytic infiltrations were also observed in the bone marrow, lymph nodes, and skin. In longitudinal sections of sciatic nerve, random, multifocal histiocytic aggregates were noted intercalating between axons, with disruption of myelin and accumulation of cellular debris (Fig 5A). Similar changes were noted in sagittal sections of the spinal cord, with disruption of neuronal tracts. Ganglia appeared unaffected. Pulmonary changes were also seen in *Asah1*^P361R/P361R^ animals, including mild oedema with scattered foamy alveolar macrophages and mild increases in neutrophils within alveolar septae. No tissue changes were noted in sections of kidneys, testes, or skeletal muscles from *Asah1*^P361R/P361R^ mice. Rare scattered vacuolation of cardiac myofibres were interpreted to be within normal limits. No intermediate phenotype was present in heterozygous animals. There was also no evidence noted of infectious disease in any of the animals examined. Safranin-O staining of femurs from *Asah1*^P361R/P361R^ mice revealed shorter epiphyseal growth plates (Fig 5B). There was also less endochondral ossification, which can explain the less intense staining representative of decreased proteoglycan levels.

Transmission electron microscopy examination indicated curvilinear tubular structures (Zarbin et al, 1985) within the lysosomal compartments of cells in hepatic and peripheral nerve sections from *Asah1*^P361R/P361R^ mice (Fig 5C) – a feature found in FD.

### Monocyte chemo-attractant protein-1 (MCP-1) production is increased in organs from *Asah1*^P361R/P361R^ mice

**Figure 6:**
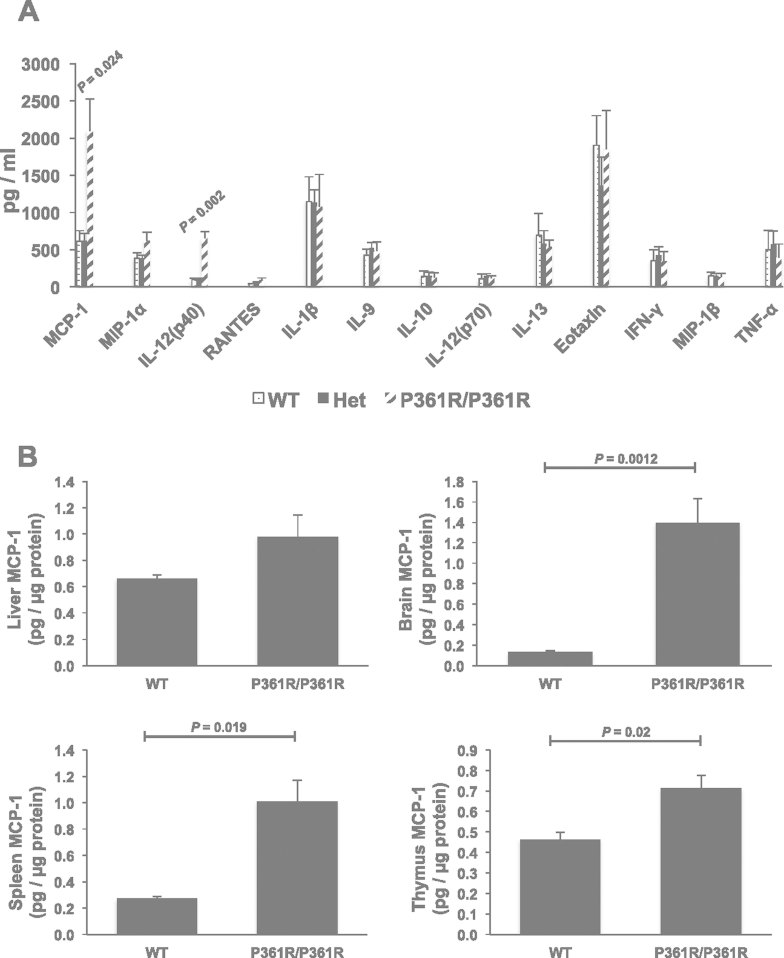
Increased levels of MCP-1 in *Asah1*^P361R/P361R^ mice Serum cytokine levels in WT (*n* = 3), heterozygotes (*n* = 5), and *Asah1*^P361R/P361R^ mice (*n* = 5). The levels of serum cytokines were measured once. Bars represent mean values. Error bars represent standard errors of the mean.MCP-1 levels in lysates from livers, brains, spleens, and thymuses of *Asah1*^P361R/P361R^ and WT mice (*n* = 4). The tissue MCP-1 levels were measured once. Bars represent mean values. Error bars represent standard errors of the mean.

We next sought to define mechanisms for the pathophysiological changes we observed in *Asah1*^P361R/P361R^ mice. As one of the most striking phenotypes of *Asah1*^P361R/P361R^ mice was the widespread macrophage infiltration, sera from WT, heterozygous, and *Asah1*^P361R/P361R^ mice at 7–9 weeks were examined for levels of various cytokines (Fig 6A). Salient differences were observed in MCP-1 and IL-12 (p40) expression; levels were found to be significantly increased in *Asah1*^P361R/P361R^ animals compared to WT mice (Fig 6A). MCP-1 (CCL2) is produced by a variety of cells and can be induced by stress. MCP-1 binds CCR2 and has both anti- and pro-inflammatory effects. To follow up on this finding, livers, brains, spleens, and thymuses were harvested from 9-week-old WT and *Asah1*^P361R/P361R^ mice. High levels of MCP-1 protein were observed in lysates from all *Asah1*^P361R/P361R^ mouse organs tested compared to those derived from WT littermates (Fig 6B). A repeat of this experiment on lysates from spleens and thymuses from a separate cohort of animals transcardially perfused with PBS also showed high levels of MCP-1 protein in *Asah1*^P361R/P361R^ mice compared to WT littermates (data not shown).

### Injection of *Asah1*^P361R/P361R^ neonates with a recombinant LV encoding human ACDase reduced the severity of the disorder

**Figure 7:**
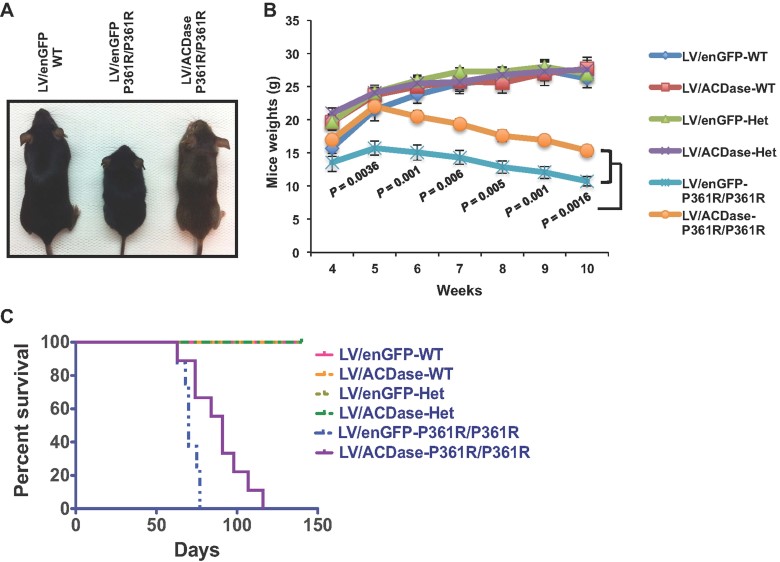
Improved growth curves and survival in *Asah1*^P361R/P361R^ mice treated as neonates with a recombinant LV engineering expression of human ACDase A representative picture of 10-week-old LV/enGFP-WT, LV/enGFP-*Asah1*^P361R/P361R^, and LV/ACDase-*Asah1*^P361R/P361R^ mice.Growth curves measured in weights *versus* age for: LV/enGFP-WT (*n* = 8); LV/ACDase-WT (*n* = 7); LV/enGFP-Het and LV/ACDase-Het (*n* = 10); LV/enGFP-*Asah1*^P361R/P361R^ (*n* = 8); and LV/ACDase-*Asah1*^P361R/P361R^ (*n* = 7). Error bars represent standard errors of the mean.Kaplan-Meier survival analysis for all treated genotypes: LV/enGFP-WT (*n* = 8); LV/ACDase-WT (*n* = 8); LV/enGFP-Het (16); LV/ACDase-Het (*n* = 34); LV/enGFP-*Asah1*^P361R/P361R^ (*n* = 8); and LV/ACDase-*Asah1*^P361R/P361R^ (*n* = 9).

We injected a total of eighty-four 1- to 3-day-old mice derived from heterozygote intercrossings via the temporal vein with LVs that engineered expression of either human ACDase (LV/ACDase) or enGFP (LV/enGFP) as a control. Fifty-one neonates each received 5 × 10^7^ IU of LV/ACDase. Genotyping after three weeks identified 9 *Asah1*^P361R/P361R^ recipients among the 51 LV/ACDase injected animals. Eight of 33 mice treated with an equivalent 5 × 10^7^ IU of LV/enGFP were also identified as *Asah1*^P361R/P361R^. Two *Asah1*^P361R/P361R^ treated animals, one heterozygote, and one WT animal in the LV/ACDase-treated group demonstrated seizures at 3 weeks and were excluded from the growth curve analysis. Physiologically, *Asah1*^P361R/P361R^ mice treated with LV/ACDase manifested intermediate phenotypes (Fig 7A). Their growth was comparable to mice from the heterozygous and WT groups until 5 weeks of age. After that point they started to lose weight at a rate similar to the LV/enGFP-treated *Asah1*^P361R/P361R^ group. That said, their body weights remained significantly higher than the weights of *Asah1*^P361R/P361R^ mice treated with control LV/enGFP (Fig 7B).

Importantly, neonatal treatment with LV/ACDase extended the lifespans of *Asah1*^P361R/P361R^ animals. All *Asah1*^P361R/P361R^ mice treated with LV/enGFP died by 11 weeks of age (median survival = 70 days) while 7 out of 9 LV/ACDase-treated mice lived beyond 11 weeks and up to 16.5 weeks (median survival = 91 days; Fig 7C). Note that two *Asah1*^P361R/P361R^ mice from the therapeutic group were euthanized at 10 weeks due to penile prolapse and an inability to urinate.

**Figure 8:**
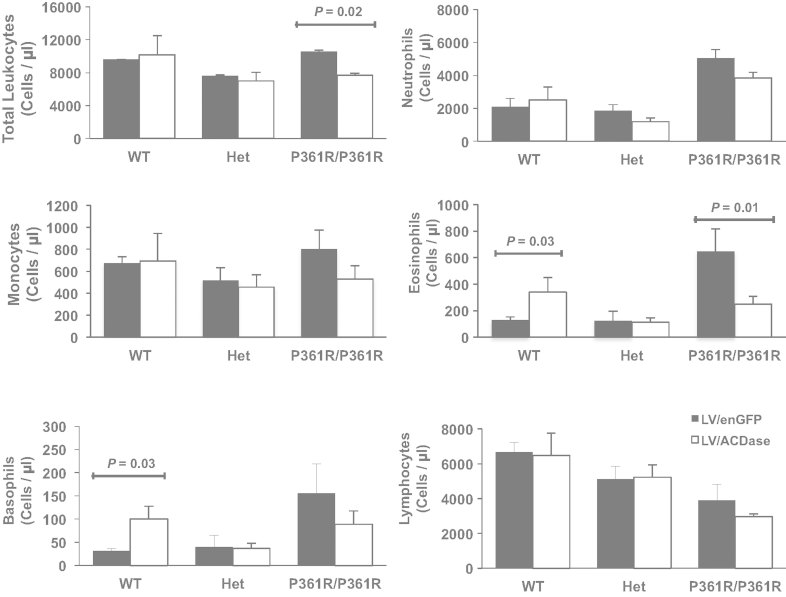
Reduced leukocyte counts in *Asah1*^P361R/P361R^ mice after LV/ACDase treatment Blood samples were collected from all animals at 10–12 weeks. Total leukocytes and differential counts were analyzed in control and experimental genotype groups: LV/enGFP-WT (*n* = 8); LV/ACDase-WT (*n* = 7); LV/enGFP-Het (*n* = 8); LV/ACDase-Het (*n* = 8); LV/enGFP-*Asah1*^P361R/P361R^ (*n* = 7); and LV/ACDase-*Asah1*^P361R/P361R^ (*n* = 9). The cell counts were measured once. Bars represent mean values. Error bars represent standard errors of the mean.

Peripheral blood analyses performed at 10–12 weeks post-treatment revealed that LV/ACDase treatment also had profound effects on peripheral blood cell counts in *Asah1*^P361R/P361R^ mice. As expected in the LV/enGFP-treated group, total leukocytes, neutrophils, monocytes, eosinophils, and basophils in *Asah1*^P361R/P361R^ mice were elevated compared to heterozygous and WT mice. In comparison, a reduction of total leukocyte counts to normal levels was observed in LV/ACDase-treated *Asah1*^P361R/P361R^ animals (Fig 8). LV/ACDase gene therapy also significantly altered the differential white blood cell profile in *Asah1*^P361R/P361R^ mice *versus* controls. Eosinophil counts showed significantly reduced levels while we observed a trend towards lower counts of monocytes, neutrophils, basophils, and lymphocytes in *Asah1*^P361R/P361R^ mice treated with LV/ACDase (Fig 8). Interestingly, we observed a trend to increased cell counts in WT mice treated with LV/ACDase compared to WT mice treated with LV/enGFP.

**Figure 9:**
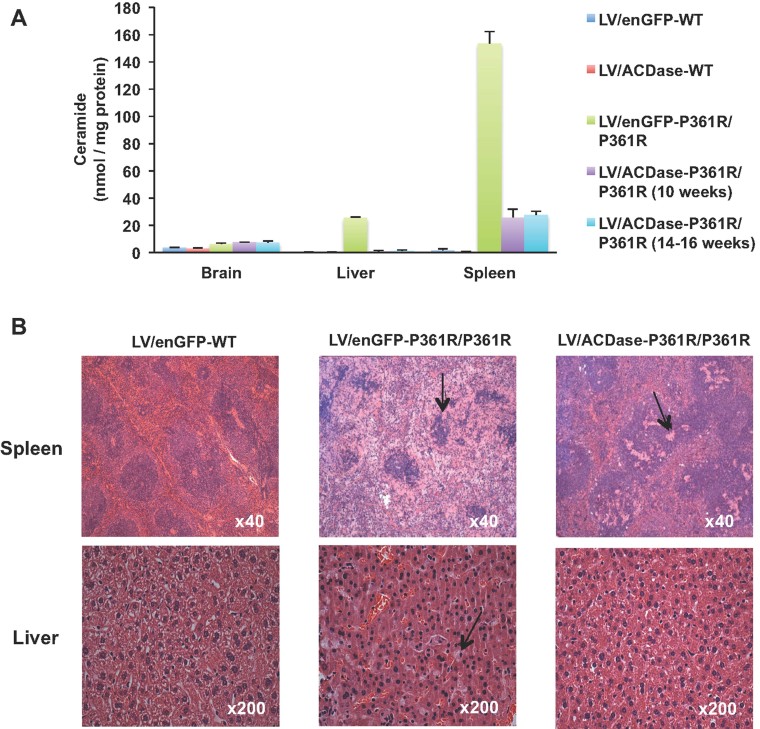
Decreased ceramide levels and diminished macrophage infiltrations in *Asah1*^P361R/P361R^ mice after LV/ACDase treatment Samples from spleens, livers, and brains of 10-week-old LV/enGFP-WT, LV/ACDase-WT, LV/enGFP-*Asah1*^P361R/P361R^, and LV/ACDase-*Asah1*^P361R/P361R^ mice, and 14- to 16-week-old LV/ACDase-*Asah1*^P361R/P361R^ mice were analyzed for total ceramide using mass spectrometry (*n* = 2 for all groups). The data points represent average values from two independent experiments. Bars represent mean values. Error bars represent standard errors of the mean.H&E staining of spleen and liver from 10-week-old LV/enGFP-WT, LV/enGFP-*Asah1*^P361R/P361R^, and LV/ACDase-*Asah1*^P361R/P361R^ mice (arrows indicate foamy macrophages).

Tissues from 10-week-old LV/enGFP-WT, LV/enGFP-*Asah1*^P361R/P361R^, and LV/ACDase-*Asah1*^P361R/P361R^ animals, along with tissues from 14- to 16-week-old LV/ACDase-*Asah1*^P361R/P361R^ mice, were assessed for total ceramide (Fig 9A and Supporting Information Fig S4A) and ceramide/DAG ratios (Supporting Information Fig S4B). Ceramide levels were strikingly reduced in the spleen and liver of *Asah1*^P361R/P361R^ mice that received a single neonatal LV/ACDase injection (Fig 9A). Total ceramide as well as ceramide/DAG ratios were reduced in the spleen and liver in the LV/ACDase-treated animals while ceramide levels in the brain remained elevated (Fig 9A). Microscopic examinations demonstrated reduction in macrophage infiltrations in the liver and spleen (Fig 9B). Mild reductions in infiltrations were also noted in the brain (data not shown). Infiltrations were still observed in the spinal cord, sciatic nerve, thymus, lymph nodes, bone marrow, lung, and skin in treated *Asah1*^P361R/P361R^ mice, however.

## DISCUSSION

ACDase has recently garnered much attention in cell biology owing to its role in cell growth and proliferation and its association with malignant transformations (Beckham et al, 2012; Kolesnick, 2002; Ogretmen and Hannun, 2004; Park and Schuchman, 2006). To this end, ACDase, its substrate ceramide, and its metabolic product sphingosine-1-phosphate (S1P) play integral roles in the tight homeostatic regulation that ultimately determines cell-fate (Loughran and Wang, 2011; Maceyka et al, 2012). Such a sphingolipid balance is particularly important during embryogenesis. Previously, homozygous ACDase “knockout” mice embryos were shown to not live beyond the four-cell stage, highlighting the importance of this enzyme in development (Eliyahu et al, 2007).

To generate a novel mammalian model of ACDase deficiency to allow us to better study FD progression, the role of lysosomal ceramide *in vivo*, and evaluate experimental therapies, we introduced a single nucleotide mutation into the murine *Asah1* coding sequence via genomic recombination (“knock-in”). We selected a mutation reported in a patient with a severe type of FD (P362R) (Li et al, 1999). Among all reported Farber patient mutations, this mutation is located in the most conserved linear amino acid region between the human and murine polypeptides. *Asah1*^P361R/P361R^ animals recapitulated features of FD and died prematurely by 13 weeks of age. Further analyses of *Asah1*^P361R/P361R^ mice revealed characteristic features of FD including low ACDase activities, high ceramide levels, histiocytic infiltrates into various organs, and the pathognomonic Farber bodies. Defects in the epiphyseal growth plate can help to explain the short limbs and growth failure we observed in *Asah1*^P361R/P361R^ mice. Furthermore, analyses of the reproductive tissue from *Asah1*^P361R/P361R^ animals support the previous finding that specific ACDase knockout in the ovaries leads to impairment of follicular development and therefore reduced fertility (Eliyahu et al, 2012). Neurological impairments in Farber patients are thought to contribute to the severity of the illness (Cvitanovic-Sojat et al, 2011); detailed neurological characterizations of the *Asah1*^P361R/P361R^ mice are now underway.

In humans, the P362R ACDase mutation results in normal levels of expression but ∼10% of WT activity (Moser et al, 1989). We note that accumulation of ceramide in *Asah1*^P361R/P361R^ mice did not trigger compensatory expression pathways as ACDase expression levels in tissues from *Asah1*^P361R/P361R^ mice were similar compared to heterozygous and WT mice (Supporting Information Fig S2). As well, the P361R mutation also did not lead to a gross reduction in ACDase protein expression and stability. To better understand how the P361R mutation may affect ACDase function, we generated a structural model of the mature enzyme based on the crystal structure of acyl-coenzyme A: isopenicillin *N*-acyltransferase (Bokhove et al, 2010) (Supporting Information Fig S5). The model predicts that murine residue P361 is located ∼20 Å away from the putative ACDase active site and unlikely to directly affect catalytic activity. Furthermore, P361 is projected to lie in a surface-exposed loop and may not affect protein folding or stability. It is plausible that the P361 residue makes up part of a protein–protein interaction interface; a mutation here may interfere with binding of co-factors such as saposin D.

Allogeneic bone marrow transplantation has shown some success in reducing the peripheral symptoms in FD (Ehlert et al, 2007); however, neurological symptoms and patient lifespans remain unaltered. Previously we demonstrated that vectors could efficiently correct the ACDase defect *in vitro* in fibroblasts (Medin et al, 1999). Corrected cells also secreted ACDase that could be taken up and used functionally by bystander cells. We later showed that recombinant LVs efficiently generate expression of ACDase both *in vitro* and *in vivo* (Ramsubir et al, 2008). Further we have recently completed experiments in enzymatically normal non-human primates (NHPs) involving autologous transplantation of LV/ACDase-transduced haematopoietic cells. There we observed supranormal levels of ACDase-specific activity in the bone marrow, peripheral blood mononuclear cells, spleen, and liver. Reductions in ceramide levels were also observed (Walia et al, 2011).

Previously we demonstrated transgene transfer into neonatal mice via direct LV injections (Yoshimitsu et al, 2004). Here we delivered the human ACDase cDNA analogously to *Asah1*^P361R/P361R^ mice in an initial attempt to correct their disease manifestations. Importantly, LV/ACDase gene transfer resulted in an intermediate phenotype in *Asah1*^P361R/P361R^ mice compared to controls as highlighted by enhanced growth, prolonged survival, reduced leukocyte counts, and lower ceramide levels. However, subsequent declines in body weights after 5 weeks still occurred and all treated mice eventually succumbed to the disease. One possibility is that human ACDase secreted by vector-transduced cells in this initial one-time therapeutic schema is not sufficient or fully accessible to all sites necessary to completely catabolize ceramide in *Asah1*^P361R/P361R^ mice.

The precise sequence of events that starts with the accumulation of ceramide and leads to widespread tissue destruction and macrophage infiltration is yet to be determined. We hypothesize that the infiltration of tissues with macrophages is mediated by chemo-attractant proteins. Indeed, serum analysis of samples from *Asah1*^P361R/P361R^ mice revealed high levels of MCP-1 protein compared to levels found in WT and heterozygous littermates. MCP-1 is a strong chemo-attractant for monocytes (Rutledge et al, 1995). High levels of MCP-1 drive monocytes from the blood circulation into sites of MCP-1 overexpression (Fuentes et al, 1995). Ceramide levels have been correlated with MCP-1 levels in adipocytes but not systemically in previous studies. Along these lines, synthetic ceramide has been shown to induce the expression of MCP-1 mRNA in adipocytes *in vitro* (Samad et al, 2006), though this may be mediated by ceramide-1-phosphate (Mitsutake et al, 2012). As well, inhibition of *de novo* ceramide synthesis in adipocytes led to a reduction in MCP-1 mRNA expression (Yang et al, 2009).

To demonstrate a direct systemic correlation, we have shown that MCP-1 levels are increased in livers, brains, spleens, and thymuses from *Asah1*^P361R/P361R^ mice. This increase in MCP-1 level was accompanied by increase in ceramide levels and infiltrations of macrophages into various organs in *Asah1*^P361R/P361R^ mice. Therefore, we suggest that the accumulation of ceramide prompts the release of a ‘call signal’ (MCP-1) that recruits circulating monocytes to help dispose of the excess ceramide from tissues. Being deficient of ACDase activity themselves, recruited macrophages fail to degrade the sphingolipids and ceramide they engulf, which results in more ceramide storage and their subsequent foamy appearance that is characteristic of FD. Note that this mechanism is in contrast to a previous model that suggested intrinsic dysregulation of leukocytes to be responsible for their abnormal behaviour in FD (Ehlert et al, 2006).

Our proposed model puts the pathophysiology of FD – a very rare disorder – in the context of a common mechanism that was shown to contribute to the pathogenesis of atherosclerosis, obesity complications, and diabetic nephropathy (Boring et al, 1998; Kanda et al, 2006; Kanamori et al, 2007). This new understanding of the disease process has impact on future therapies for FD. Indeed at every level the ceramide, MCP-1, and macrophage axis can be explored as a potential treatment modality for Farber disease.

In conclusion, we have shown that our novel animal model of ACDase deficiency accurately recapitulates the FD phenotype and can be utilized to evaluate novel experimental therapies. We also suggest a new mechanistic viewpoint that helps to explain the disease process and provides a platform for developing new treatment modalities. Moreover, this model of ACDase deficiency can also be utilized to examine sphingolipid metabolism and signalling along with their roles in cancer biology and haematopoiesis.

## MATERIALS AND METHODS

### Gene targeting of *Asah1* gene and mice breeding

Two arms of homology were cloned into ploxPneo-1 plasmid, which has a neomycin resistance gene (neo^R^) flanked by two loxP sites (Supporting Information Fig S1A). The homologous arms were amplified by PCR using genomic DNA from the W4/129Sv mouse strain as a template.

The long arm was 4-kb long and consisted of a genomic fragment extending from exon 8 to 12 with flanking intron sequences (Supporting Information Fig S1B). The amplified fragment was cloned into ploxPneo-1 plasmid downstream of the neo^R^ cassette. The short arm was 2.4 kb long spanning from exon 13 and the adjacent intronic region to exon 14 (Supporting Information Fig S1B). We amplified this arm and then modified it by introducing a single nucleotide mutation involving the replacement of cytosine with guanine on exon 13 in the location that corresponds to position number 1082 of the murine cDNA. This mutation leads to the replacement of proline with arginine at position 361 of the murine polypeptide. The vector was designed so that the neo^R^ cassette occupies an intronic region between exons 12 and 13. The construct was confirmed by restriction digestion and DNA sequencing. The targeting vector was linearized and transfected into W4 mouse ES cells by electroporation. We then screened 700 ES-cell clones that survived antibiotic selection. Southern blot analyses on Hind III-digested genomic DNA revealed one positive clone for homologous recombination (Supporting Information Fig S6). Additional validation procedures included PCR amplification of the neo^R^ amplicon in the ES cells (data not shown), sequencing of ES cell genomic DNA and tissue cDNA (data not shown), and FISH analysis (Supporting Information Fig S7). Targeted W4 ES-cells were aggregated with CD1 mouse morulae, using a standard aggregation method, to generate chimeric mice (Nagy et al, 2003). Crossing the chimera with CD1 mice resulted in progenies heterozygous for the mutation. To generate *Asah1*^P361R/P361R^ mice, we intercrossed heterozygotes. All animal procedures were approved by the UHN Animal Care Committee.

### Ovary analyses

WT, heterozygous, and *Asah1*^P361R/P361R^ female mice were euthanized at 7 weeks. Ovaries were harvested and analyzed as described previously (Eliyahu et al, 2012).

### FISH analysis

BAC clones RP23-478E13 and RP23-117M15 corresponding to the *Asah1* gene and RP23-180H13 and RP23-2E9 corresponding to the *Lamp1* gene, respectively, were obtained from the Toronto Centre for Applied Genomics and labeled using SpO (*AsahI*) or SpG (*Lamp1*) with a nick translation kit (Abbott Molecular, Des Plaines, IL). FFPE 4 µm kidney sections were deparaffinized in xylene, dehydrated in ethanol, pretreated in citrate buffer (pH 6.8), digested with pepsin, and then denatured. Slides were hybridized for 2 days, washed, and counterstained with DAPI (Vector Laboratories, Burlingame, CA). Specimens were examined at 63× magnification on an Imager M1 Zeiss microscope (Carl Zeiss Canada Limited). The JAI CV-M4 + CL progressive scan monochrome camera (JAI Inc., San Jose, USA) and MetaSystems Isis FISH Imaging software v5.3 (MetaSystems, Germany) were used to capture images.

### Assessment of ACDase activity in intact mouse embryonic fibroblasts (MEFs)

MEFs were generated according to the method of Theunissen and Petrini (2006). ACDase activity was assessed *in situ*, in intact living MEFs, as previously reported (Levade et al, 1996). This method allows specific determination of lysosomal acid sphingomyelinase and ACDase activities using a natural radiolabelled sphingomyelin as substrate.

### Quantitation of ceramide

After extraction of lipids, ceramide levels were determined using *E. coli* diacylglycerol kinase and (γ^32^P)-ATP as previously described (Bielawska et al, 2001). Ceramide levels were also determined using mass spectrometry as described previously (Munoz-Olaya et al, 2008).

### Acid ceramidase protein expression in various organs

Kidneys, livers, and spleens were collected from three *Asah1*^P361R/P361R^ mice, three WT, and three heterozygous littermates and lysed via RIPA lysis buffer (Thermo Scientific, Waltham, MA, USA) and sonication. 50 µg of protein was loaded on 10% SDS–PAGE and then transferred onto a PVDF membrane (Thermo Scientific, Waltham, MA, USA) via semi-dry transfer. Following the transfer, the membranes were blocked overnight at 4°C with 5% non-fat dried milk (NFDM). The following primary antibodies were used at the specified dilutions in 2.5% NFDM/TPBS: anti-β-actin (Millipore, Billerica, MA, USA – 1:1000) and anti-ACDase (Sigma–Aldrich, St. Louis, MO, USA – 1:1000). The following secondary antibodies were used in 2.5% NFDM/TPBS: anti-rabbit IgG (Thermo Scientific, Waltham, MA, USA) and anti-mouse IgG-HRP (GE Healthcare, Waukesha, WI, USA). Membranes were developed using ECL (Thermo Scientific Pierce, Waltham, MA, USA) and exposed onto autoradiograph film. Subsequent densitometry was performed using Image J v1.44 (National Institute of Health, Bethesda, MD, USA).

### Liposome-based assay to measure ACDase activity

Protein extracts were prepared by sonication of tissues in a 0.25 M sucrose solution. Protein concentrations were determined by Coomassie Protein assay (Thermo Scientific, Rockford, IL, USA); protein extracts were diluted with 0.25 M sucrose to the desired concentration. Liposome composition was 500 µM phosphatidylcholine (Avanti Polar Lipids, Alabaster, AL, USA), 450 µM bis-monoacylglycero-phosphate (BMP; Avanti Polar Lipids, Alabaster, AL, USA), 50µM cholesterol (Avanti Polar Lipids), and 25 µM C12-NBD-Ceramide (Cayman Chemical, Ann Arbor, MI, USA). Liposomes were prepared by sonication in 200 mM phosphate/citrate buffer (pH 4.6) with the resultant liposome radius determined by DLS and found to be within the 30–40 nm range. Liposome solution and protein extracts were then mixed with purified SapD (Popovic and Prive, 2008) to yield the final reaction mixture: 0.5 mg/ml protein, 0.25 µg/ml SapD, and 12.5 µM C12-NBD-Ceramide. The reactions were incubated at 37°C with agitation for 3 h, and then stopped by addition of 100 µl of chloroform:methanol (1:1). After vortexing and centrifugation, the bottom chloroform layer was extracted and dried by SpeedVac. The dried lipids were then resuspended in 20 µl of chloroform:methanol (2:1), and appropriate amounts of the organic solution was spotted on a silica gel 60 thin-layer chromatography (TLC) plate (EMD Millipore, Billerica, MA, USA). Lipids were separated by using chloroform:methanol:ammonia (90:20:4) and visualized by a PhosphorImager (Storm 840). The fluorescence signals were quantified by ImageQuant (Molecular Dynamics, Sunnyvale, CA, USA).

**The paper explained**

PROBLEM:

Acid ceramidase (ACDase) is a key enzyme in regulating sphingolipid metabolism. It is a rheostat in controlling the balance between the substrate ceramide – an apoptotic molecule – and the phosphorylated product sphingosine-1-phosphate, which has mitogenic properties. The deregulation of this enzyme has been reported in a number of malignancies as well as chronic diseases. Furthermore, the inherited deficiency of this enzyme is associated with Farber disease (FD), a severe and fatal lysosomal storage disorder. A previous attempt to generate an ACDase knockout mouse resulted in lethal homozygous phenotype indicating the crucial role of this enzyme in development. The lack of an animal model of ACDase deficiency has become a challenge to advance the research on FD, ACDase, and sphingolipid metabolism.

RESULTS:

In this study, we generated a mouse model that harbors a single-nucleotide mutation in ACDase gene (*Asah1*). Our analyses of the phenotype showed characteristic features of FD in homozygous animals. Affected mice manifested growth retardation and died by the age of 13 weeks. Various tissues demonstrated reduced enzyme activities, accumulation of ceramide, and abnormal infiltrations with foamy macrophages. We also could identify the diagnostic curvilinear structures – Farber bodies – under the electron microscope. Lentivector-mediated neonatal gene delivery of human ACDase cDNA partially corrected the disease manifestations. Importantly, we propose in this study a new mechanism that links the build-up of ceramide with the phenotype of the disease (macrophage infiltrates) through MCP-1 overexpression.

IMPACT:

We present the first viable animal model of systemic ACDase deficiency. As far as FD is concerned, this model helps understand the pathophysiology of this disorder and serves as a platform to test and develop new treatment modalities for the management of this fatal disease. This model will also facilitate studying the effects of ACDase deficiency on various cellular functions. In this respect, this model can be utilized to understand the effects of ceramide storage in different tissues and organ environments.

### Peripheral blood and cytokine analysis

Peripheral blood was collected from saphenous veins or from cardiac punctures into EDTA-coated tubes. Peripheral blood counts including total leukocyte, neutrophil, monocyte, eosinophil, basophil, and lymphocyte counts were analyzed by Hemavet (Drew Scientific Group, Waterbury, CT, USA). Serum cytokine levels were analysed using BioRad multiplex cytokine magnetic plate assay (BioRad). Tissues for MCP-1 measurements were lysed using ultrasound and total protein levels were measured by Bradford protein assay (BioRad). MCP-1 levels were measured by ELISA (BioRad).

### Histopathology and ultrastructural analysis

For histological analyses, WT, heterozygous, and *Asah1*^P361R/P361R^ mice were euthanized by CO_2_ inhalation and tissues were collected and fixed in 10% neutral-buffered formalin, embedded in paraffin, and sectioned and stained with haematoxylin and eosin (H&E). Specimens were examined and scored without advance knowledge of genotype or treatment. For Safranin-O staining, WT and *Asah1*^P361R/P361R^ mice were euthanized at 7 weeks. Dissected limbs were fixed in 10% formalin then decalcified in formical-4TM (Decal Chemical Corporation, Suffern, NY, USA) for 3 days before embedding in paraffin wax. Sections (4–5 µm) were stained with safranin-O. Slides were visualized and photographed with a confocal laser-scanning microscope (Carl Zeiss 510 Meta).

For electron microscopy, *Asah1*^P361R/P361R^ mice were euthanized by CO_2_ inhalation and tissues were collected and fixed with 1% glutaraldehyde/ 3.2% paraformaldehyde in 0.1 M phosphate buffer for 24 h. The specimens were then post-fixed in 1% OsO_4_ for 1 h. They were then dehydrated in acetone solution and embedded in epoxy resin. Ultrathin sections were cut and stained with uranyl acetate before examination in a transmission electron microscope.

### Lentivirus (LV) production and neonatal injections

LVs pseudotyped with vesicular stomatitis virus-glycoprotein (VSV-g) were generated from the transient co-transfection of the SIN transfer vector (pHR′-EGFP.WPRE or pHR′-huACDase.IRES.huCD25.WPRE) (Ramsubir et al, 2008), second-generation LV packaging construct pCMVΔ8.91, envelope plasmid pMD.G, and pAdVAntage™ plasmid (Promega, Madison, WI, USA) into HEK 293T cells (ATCC, Manassas, Virginia, USA) using the polymer polyethyleneimine (PEI) as described previously (Amarnath et al, 2011). Virus supernatants were harvested 48 and 72 h after transfection, filtered, and sterilized using 0.22-µm filter (Nalgene, Rochester, NY, USA), and concentrated at 50,000*g* for 2 h using an Optima L-100 XP Ultracentrifuge (Beckman Coulter, Mississauga, Ontario, Canada). LV pellets were re-suspended in serum-free RPMI-1640 medium (Sigma–Aldrich). Functional titres were determined by serially-diluted LV transductions of HEK 293T cells, followed by subsequent flow cytometry analyses. One- to 3-day-old mice were injected intravenously via the temporal vein as described (Sands and Barker, 1999). All mice received 5 × 10^7^ IU of either LV/ACDase or LV/enGFP.

### Generation of a mouse ACDase variant structural model

A structural model of murine ACDase (P361R) was generated using the I-TASSER server (Zhang, 2008), which identified acyl-coenzyme A: isopenicillin *N*-acyltransferase (PDB ID 2 × 1E) as one of the top structural homologs. Active site residues in murine ACDase were predicted from a structure based sequence alignment with acyl-coenzyme A: isopenicillin *N*-acyltransferase and mutagenesis data from human ACDase (Shtraizent et al, 2008).

### Statistics

Data are presented as mean results ± standard error of the mean, as per figure legends. Statistical analyses were performed using two-tailed, unpaired Student's *t*-tests with unequal variances. Statistical analyses of survival data were performed using the Mantel-Cox log-rank test (GraphPad Prism, GraphPad Software, San Diego, CA). *p*-Values <0.05 were considered significant.

## Author contributions

The study was conceived by JAM. AMA and JAM designed the study. AMA, TL, and JAM analyzed the data and wrote the manuscript. AMA performed most of the experiments. AMA, SEG, and RH designed the targeting vector and the “knock-in” experiments. SC, VG, and TL analyzed the tissues for ceramide levels and ceramide/DAG ratios. JC and GF performed the mass spectrometry analyses of ceramide. PVT conducted the histopathology examinations. AMA and SD did the electron microscopy examinations. AMA and MJE analyzed the tissues for MCP-1 levels by ELISA. KNK, ZJX, and GGP generated the three-dimensional structure of the mutated ACDase enzyme and performed the liposome assay. CMS and EHS performed the analyses on the ovaries. AMA and JCMW conducted the gene delivery experiment with contributions from BCYA.

## For more information

OMIM database entry for ACDase: http://www.omim.org/entry/613468

The Online Metabolic & Molecular Bases of Inherited Disease for Farber Disease: http://www.ommbid.com/OMMBID/the_online_metabolic_and_molecular_bases_of_inherited_disease/b/abstract/part16/ch143

I-TASSER server: http://zhanglab.ccmb.med.umich.edu/I-TASSER/

Medin lab: http://www.uhnresearch.ca/researchers/profile.php?lookup=4071

## Supplementary material

As a service to our authors and readers, this journal provides supporting information supplied by the authors. Such materials are peer reviewed and may be re-organized for online delivery, but are not copy-edited or typeset. Technical support issues arising from supporting information (other than missing files) should be addressed to the authors.
