# Supplementary material for: Systemic ceramide accumulation leads to severe and varied pathological consequences
Source: EMBO Mol Med. 2013 May 16;5(6):827–42. doi: 10.1002/emmm.201202301 (PMC3779446; doi:10.1002/emmm.201202301)
Supplement: Supplementary file 1 [file emmm0005-0827-SD1.pdf]

EMBO
Molecular Medicine

Systemic Ceramide Accumulation Leads to Severe and Varied Pathological Consequences

Abdulfatah M. Alayoubi, James C.M. Wang, Bryan C.Y. Au, Stéphane Carpentier, Virginie Garcia, Shaalee Dworski, Samah El Ghamrasni, Kevin N. Kirouac, Mathilde J. Exertier, Zi Jian Xiong, Gilbert G. Privé, Calogera M. Simonaro, Josefina Casas, Gemma Fabrias, Edward H. Schuchman, Patricia V. Turner, Razqallah Hakem, Thierry Levade, Jeffrey A. Medin

Corresponding author: Jeffrey Medin, University Health Network

Review timeline:

Submission date:	28 November 2012
Editorial Decision:	19 December 2012
Revision received:	17 March 2013
Editorial Decision:	20 March 2013
Revision received:	04 April 2013
Accepted:	05 April 2013

Transaction Report:

(Note: With the exception of the correction of typographical or spelling errors that could be a source of ambiguity, letters and reports are not edited. The original formatting of letters and referee reports may not be reflected in this compilation.)

Editor: Roberto Buccione

1st Editorial Decision 19 December 2012

Thank you for the submission of your manuscript to EMBO Molecular Medicine.

We have now heard back from the three referees whom we asked to evaluate your manuscript.

You will see that in general the Reviewers are supportive of your work and underline its considerable potential interest. They do however, raise a number of specific concerns that require your action before we can consider your manuscript for publication.

Reviewer 1 praises the quality of your work but is concerned that the level of insight into the pathophysiology of Faber disease is limited. While we believe that to extend this part of your work may not be necessary, I nonetheless strongly encourage you to develop the study as far as realistically possible in this direction to strengthen your manuscript and increase its overall impact. Reviewer 1 also asks specific questions that need to be addressed.

Reviewer 2 details a number of specific issues concerning experimental and/or textual aspects. For instance, s/he notes a number of inconsistencies or insufficient support for some conclusions. I also note that Reviewer 2, similarly to Reviewer 1, encourages further analysis of the disease phenotype and would like to see an improved behavioural and functional characterisation. I strongly advise you to give each item careful consideration.

Reviewer 3 expresses concern on a number of issues spanning from proper statistical analysis to

experimental inconsistencies that need proper resolution. S/he also mentions certain unclear or ambiguous statements that also require your action. Finally, I note that Reviewer 3 specifically questions your observation on hydrocephalus, and similarly to Reviewer 2, would like exhaustive clarification on this issue.

While publication of the paper cannot be considered at this stage, we would be pleased to consider a suitably revised submission, provided, however, that the Reviewers' concerns are addressed with additional experimental data where appropriate.

Please note that it is EMBO Molecular Medicine policy to allow a single round of revision only and that, therefore, acceptance or rejection of the manuscript will depend on the completeness of your responses included in the next, final version of the manuscript.

As you know, EMBO Molecular Medicine has a "scooping protection" policy, whereby similar findings that are published by others during review or revision are not a criterion for rejection. However, I do ask you to get in touch with us after three months if you have not completed your revision, to update us on the status. Please also contact us as soon as possible if similar work is published elsewhere.

I look forward to receiving your revised manuscript as soon as possible.

***** Reviewer's comments *****

Referee #1 (Comments on Novelty/Model System):

This study presents a new and viable mouse model of Faber disease. The technical quality of the data is excellent and the mice will be very useful to the medical community studying Farber disease

Referee #1 (General Remarks):

This is an excellent study describing the generation of the first viable Farber disease mouse model. The study was performed at a high level. Having said that, it does not give any new mechanistic insight into how ceramide accumulation leads to FD pathophysiology, and for that reason, the study might be considered a little preliminary and somewhat descriptive

I only have two additional comments:

1. What were the levels of MCP-1 after ACDase treatment?
2. Was ACDase activity measured in the mouse?

Referee #2 (Comments on Novelty/Model System):

This manuscript describes the development of a mouse model of Farber disease. The mouse model appears to mimic the human disease in virtually every aspect measured. Therefore, it will become an outstanding model to disect the disease pathogenesis and develop effective therapeutic interventions for this disease.

Referee #2 (General Remarks):

Alayoubi et al., describe the creation, characterization, and initial attempt at treatment of the mouse model of Farber disease. Several previous attempts to make this model have failed. Therefore, the investigators "knocked-in" a human mutation known to cause a relatively severe form of Farber disease. The investigators examined the body weight, life span, ceramide accumulation, hematologic parameters, and performed a histological survey. The resultant homozygous mutant mouse is viable and has a biochemical, visceral, skeletal, and hematological phenotype that is typical of Farber disease. Finally, the investigators determined the efficacy of intravenous delivery of a recombinant lentiviral vector encoding the human acid ceramidase cDNA to affected newborn Farber mice. The

treated mice had a significant increase in body weight and life span as well as a decrease in ceramide accumulation in systemic tissues.
The significance of this study is increased due to the difficulty in generating this mouse model of human disease. In addition, this is a relatively thorough initial characterization of this model. Finally, although the treated mice still succumb to the disease, the authors make an initial attempt at treating this disease. This is an important and relatively faithful model of Farber disease and will be invaluable for the dissection of the disease pathogenesis and development of effective therapies.

Major Comments:

1) The authors mention that the mutation was chosen due to the fact that it resulted in a relatively severe form of Farber disease. They also mention that the human mutation did not decrease the expression of acid ceramidase but diminished enzyme activity to less than 10%. The authors show in the Supplemental Figures that the mutated acid ceramidase was still expressed at relatively high levels (Western blot) but they do not show the level of enzyme activity. This would be helpful to more thoroughly understand the mouse model and murine disease.
2) The MRI data showing hydrocephalus in an affected animal is premature. Although this is something of a technical tour-de-force, it is not clear if these data are relevant to the murine disease. It is a single animal and some mouse strains are prone to hydrocephalus. These data should be removed from the paper unless the authors can show a statistically significant increase in hydrocephalus in a larger cohort of affected animals.
3) Although the authors clearly demonstrate that the nervous system is affected in this model, the analysis is a bit superficial. They also mention that the animals have defects in grip strength but show no data. The authors mention that a more thorough characterization of the CNS disease is underway. Hopefully, that study will include detailed regional (CNS and PNS) histomorphology, biochemical analyses, and a thorough behavioral/functional characterization.

Minor Comments:

1) Were the mice transcardially perfused prior to collecting the various tissues for cytokine analysis? If so, the authors should mention this in the Materials and Methods. If not, this becomes problematic due to the increased cytokine levels in the serum.
2) Figure 4 refers to two methods, A and B, but only shows one data set.
3) Similarly, Figure 7 shows tissue ceramide levels for untreated and treated WT and Farber mice. Apparently, panels E and F show data from two independent methods. Showing both data sets is not necessary since the data appear to be virtually identical. The larger separate graphs (7E) demonstrate the data in a more convincing and understandable way. In addition, the text on pg 11 refers to the histology in Figure 7F. This will be correct if the authors delete the current Figure 7F (single graph, ceramide levels).
4) The arrows in Figures 5 and 7G are too small to see.
5) Although the authors show the life span curves for various groups of untreated and treated mice, they should formally state the exact median life spans in the text or Figure legends.

Referee #3 (Comments on Novelty/Model System):

The use of standard error of the mean with an N=2 is not appropriate. Clarifying between which groups significance has been achieved is important and measuring not only if treated are different from untreated but also how close treated are to normal would be helpful.

Referee #3 (General Remarks):

Symptoms are subjective. Only humans can describe symptoms. Animals have clinical signs.

What is general dystrophy? What is pelvic prolapse and does it occur in patients? In males?

It is not clear why the ovaries were examined so closely? "Furthermore, analyses of the ovaries from Asah1P361R/P361R animals support the previous finding that ACDase knockout leads to impairment of follicular development and therefore reduced fertility (Eliyahu et al., 2012)." But it is

stated that KO mice did not survive beyond 4 cells.

Mice cannot be "covered in fat". Please describe what is seen. Are alterations in fat distribution seen in human patients?

Hydrocephalus was seen in only one of two mice so the legend for figure 1: "Hydrocephaly was detected in the Asah1P361R/P361R mouse" needs to be more precise - Hydrocephaly was detected in an Asah1P361R/P361R mouse. Is hydrocephalus seen in human FB patients?

Page 6 - RBCs and hemoglobin were not only "slightly increased" but were significantly (P=<0.05) increased based on Figure 3B.

Where was the ceramide in the heart if "Rare scattered vacuolation of cardiac myofibers were interpreted to be within normal limits" but "total ceramide levels were dramatically elevated"?

Is there a toxic concern with lentivirus therapy as "Two Asah1P361R/P361R treated animals, one heterozygote, and one WT animal in the LV/ACDase-treated group demonstrated seizures at 3 weeks and were excluded from the
growth curve analysis."? This should be discussed.

Describe more specifically the characteristics that defined the treated mice as "generally appeared healthier".

While an increase in lifespan (when not euthanized at 10 weeks for pelvic prolapse) to 16.5 weeks is significant, it is still only a 50% increase in lifespan with therapy. Would this mean that children who die at two years might be expected to live until three? Is it know why they (children or mice) die?

For the various graphs, it appears the statistics that are significant are between RV/en-treated and RV/ACDase-treated mice (as the P-value lines connect those two groups). Shouldn't differences or similarities be evaluated between treated and WT to determine how close (or not) the improvements have been towards normal?

It is not clear which groups are being evaluated in the body weight of Fig 7B. It would appear there are no differences between the LV/ACDase- and LV/enGFP-treated affected mice beyond 5 weeks (as stated in the text) but there are P=<0.001 indications for 9 and 10 weeks - that looks like it is between WT and treated.

Is the standard error of the mean really statistically informative with an N=2 (Figure 4)?

Figure 7D: Why do the authors postulate that basophils and esosinophils go up with LV/ACDase treatment in WT mice? Are lymphocytes now significantly lower than normal in LV-treated mice? They look lower in Fig. 7D but no statistical significance is indicated.

Figure 7E would be improved by having WT and affected values for those not treated with any LV because the scale in Figure 4 is nmol/mg and in Figure 7E is pmol/mg. It would be best if the scales were the same. Figure 7F uses nmol/mg.

Figure 7G: the arrows are very small and hard to see especially in the two spleen pictures.

"Microscopic examinations demonstrated reduction in macrophage infiltrations in the liver and spleen (Fig. 7F)" is actually Fig. 7G.

In which tissues is the vector integrating - either actually in these experiments or from previous experiments?

Discussion: "We have recently completed experiments in enzymatically-normal non-human primates (NHPs) involving autologous transplantation of LV/ACDase-transduced hematopoietic cells. There we observed
supranormal levels of ACDase-specific activity in the bone marrow, peripheral blood mononuclear

cells, spleen, and liver. Reductions in ceramide levels were also observed (Walia et al., 2011)." Does this mean that ceramide levels in normal NHPs were reduced to below normal?

As you postulate that the failure to completely reverse the phenotype may be due to the human cDNA, are experiments planned or underway to use the mouse cDNA?

How confident are the authors that the tissue destruction ("...widespread tissue destruction by lipid-laden macrophages...") is caused by macrophages and that it is not simply that macrophages are present as a response to tissue destruction and are there to scavenge necrotic tissue, which is a normal macrophage function?

1st Revision - authors' response 17 March 2013

We have modified our submission to comply with all the Reviewer's suggestions. In this respect we have done different ACDase assays on cell and tissue extracts and have imaged many more animals (both homozygotes and controls) by MRI to confirm the massive hydrocephaly in the Farber mice that we previously identified. We also measured MCP-1 levels in various tissues from homozygous and control animals after lentivector treatment. Textual/presentation suggestions have also all been addressed. Following, we provide point-by-point responses to all the Reviewer's comments.

Response to Reviewer #1

This is an excellent study describing the generation of the first viable Farber disease mouse model. The study was performed at a high level. Having said that, it does not give any new mechanistic insight into how ceramide accumulation leads to FD pathophysiology, and for that reason, the study might be considered a little preliminary and somewhat descriptive.

Response: Our study suggests that some features of massive tissue accumulation of ceramide and the pathophysiology of FD are related to the increased production of chemokines such as MCP-1/CCL2, a mechanism never described so far in the context of FD.

1) What were the levels of MCP-1 after ACDase treatment?

Response: We have gone back and measured by ELISA the levels of MCP-1 protein in archived tissues from WT and *Asah1*$^{P361R/P361R}$ animals treated with either LV/enGFP or LV/ACDase.

Results: See Figure (following): LV/ACDase treatment resulted in a statistically significant reduction of MCP-1 levels in the liver. A trend towards lower levels of MCP-1 protein was observed in both the brain and thymus. MCP-1 levels were not reduced in the treated spleen. One caveat: The mice were not perfused prior to collecting the organs. MCP-1 in the serum may thus have contributed to the levels measured in the organs of these archived tissues - though reductions in treated animals may thus reflect systemic decreases due to the LV treatment.

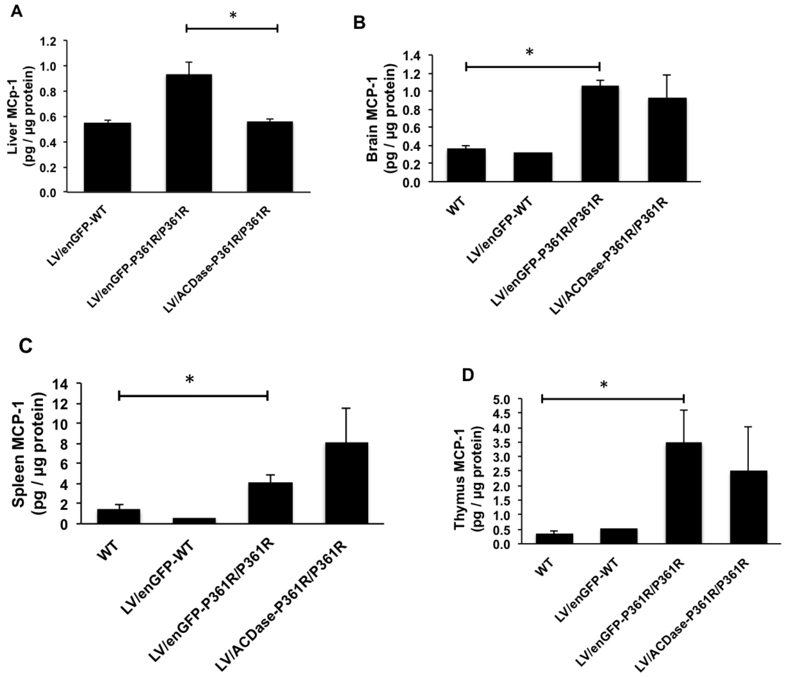

Figure. Reduced levels of MCP-1 in tissues from *Asah1*$^{P361R/P361R}$ mice treated as neonates with a recombinant LV/ACDase. (*A*) MCP-1 levels in livers from LV/enGFP-WT (n=2), LV/enGFP-*Asah1*$^{P361R/P361R}$ (n=3), and LV/ACDase-*Asah1*$^{P361R/P361R}$ mice (n=4). (*B*) MCP-1 levels in brains from WT (n=4), LV/enGFP-WT (n=1), LV/enGFP-*Asah1*$^{P361R/P361R}$ (n=3), and LV/ACDase-*Asah1*$^{P361R/P361R}$ mice (n=5). (*C*) MCP-1 levels in spleens from WT (n=4), LV/enGFP-WT (n=1), LV/enGFP-*Asah1*$^{P361R/P361R}$ (n=3), and LV/ACDase-*Asah1*$^{P361R/P361R}$ mice (n=3). (*D*) MCP-1 levels in thymuses from WT (n=4), LV/enGFP-WT (n=1), LV/enGFP-*Asah1*$^{P361R/P361R}$ (n=3), and LV/ACDase-*Asah1*$^{P361R/P361R}$ mice (n=4). Bars represent mean values. Error bars represent standard errors of the mean. $^{*}P < 0.05$ for groups indicated.

2) Was ACDase activity measured in the mouse?

Response: Yes it was, using different methods.

We based the overall idea on generating this 'knock-in' model of ACDase deficiency on the description of a patient (the only such patient identified in the world and reported to date with this mutation) and the results of one of our co-authors (Dr. Schuchman) using plasmid transfection studies to demonstrate that this unique human mutation (in a region that is highly conserved between mouse and human – though not in the active site, as we show in the modelling figure in our manuscript; Supplemental Fig. S5) – leads to reduced ACDase activity (Li et al., 1999 Genomics 62:223-231).

Results: One method we tested employed a radiolabelled, natural substrate, i.e. [^{3}H-fatty acid-labelled]ceramide. This assay, described by Dulaney and Moser (in Glew R.H. and Peters S.P., eds., Practical Enzymology of the Sphingolipidoses, 1977, Alan R. Liss Inc., New York, pp. 283-296), which is an *in vitro* assay in the presence of a detergent mixture, was carried out using both liver and kidney samples from the mouse and controls. As shown in the Table (following), ACDase activity was consistently reduced, but not totally deficient, in both liver and kidneys from homozygous mice. Partial reduction was seen in heterozygotes.

Table: *In vitro* ACDase activity determined in liver and kidney lysates from Asah1$^{P361R/P361R}$ mice and controls. Enzyme activity was determined at least in duplicate in all samples using varying protein amounts (0.25 to 2 mg) and radiolabelled ceramide as the substrate.

mouse	number of independent determinations (each done in duplicate at least)	mean liver enzyme activity (nmol/h.mg protein)	mean kidney enzyme activity (nmol/h.mg protein)
wild-type 33	2	1.46	2.08
wild-type 34	4	2.10	3.05
heterozygote 21	2	1.00	1.86
heterozygote 29	2	1.56	1.68
homozygote 20	4	0.83	1.39
homozygote 59	2	0.74	0.95

In a second series of experiments, ACDase activity was assessed *in situ*, i.e., in intact living cells, as previously reported (Levade et al. J Lipid Res. 1996; 37(12):2525-38). This method allows specific determination of lysosomal acid sphingomyelinase and ACDase activities using a natural radiolabelled sphingomyelin as substrate. We used MEFs isolated from WT, heterozygous, and homozygous Asah1$^{P361R/P361R}$ mouse embryos. Living cells were loaded with [^{3}H-ceramide]-labelled sphingomyelin for 40 hours. Lipids were then extracted, separated by thin-layer chromatography, and quantified by liquid scintillation counting after scraping. Data clearly showed deficient ACDase activity in homozygous MEFs as evidenced by accumulation of lysosomal ceramide: whereas undegraded ceramide represented 22% of sphingomyelin metabolites in WT MEFs and 30% in heterozygous MEFs, it accounted for 62% and 68% in two distinct homozygous Asah1$^{P361R/P361R}$ MEF cell lines. In a separate repeat experiment, undegraded ceramide represented 81% and 86% in two distinct homozygous Asah1$^{P361R/P361R}$ MEF cell lines as compared to only 10% in WT MEFs. Of note, degradation of sphingomyelin in homozygous Asah1$^{P361R/P361R}$ MEFs was normal. The latter data establish the functional deficiency of murine P361R mutant ACDase. These data have been added to the text in the Results Section of the Revised Manuscript.

In a third set of experiments, ACDase activity in lysates from various organs of mutant and control mice was evaluated using a liposome-based assay. In this *in vitro* enzyme assay, the substrate (a long-chain fluorescent analog of ceramide) is incubated with the enzyme in the absence of detergents but in the presence of acidic phospholipids (i.e. BMP) and the ACDase activator, i.e. saposin D. In all organs tested (liver, heart, spleen, thymus, and brains), ACDase activities were significantly reduced in lysates derived from homozygous *Asah1*$^{P361R/P361R}$ tissues compared to samples from WT animals. These data have also been added to the manuscript (new Figure 4B).

Lastly, it is very clear from our data (manuscript Figure 4) that the Asah1$^{P361R/P361R}$ mice accumulate substantial levels of ceramide and faithfully recapitulate Farber Disease – even down to the presence of the diagnostic Farber bodies in cells (Figure 5C). Addition of the wild-type ACDase cDNA through LV-mediated gene transfer specifically reduced ceramide accumulation in comparison to control vector administration and improved outcomes (Figure 7E).

Response to Reviewer #2

Major Comments:

1) The authors mention that the mutation was chosen due to the fact that it resulted in a relatively severe form of Farber disease. They also mention that the human mutation did not decrease the expression of acid ceramidase but diminished enzyme activity to less than 10%. The authors show in the Supplemental Figures that the mutated acid ceramidase was still expressed at relatively high

levels (Western blot) but they do not show the level of enzyme activity. This would be helpful to more thoroughly understand the mouse model and murine disease.

Response: Please see comments above for Referee #1.

2) The MRI data showing hydrocephalus in an affected animal is premature. Although this is something of a technical tour-de-force, it is not clear if these data are relevant to the murine disease. It is a single animal and some mouse strains are prone to hydrocephalus. These data should be removed from the paper unless the authors can show a statistically significant increase in hydrocephalus in a larger cohort of affected animals.

Response: We imaged 5 WT and 5 more homozygous *Asah1*$^{P361R/P361R}$ animals by MRI to further investigate this finding. Four additional homozygous mice showed massive hydrocephaly (see Figure 1F and following Fig). WT animals were unremarkable. In total, 5 out of 7 homozygous animals manifested hydrocephaly. Five WT and 2 heterozygous mice were unremarkable. <u>The text and figure legend have been revised accordingly.</u>

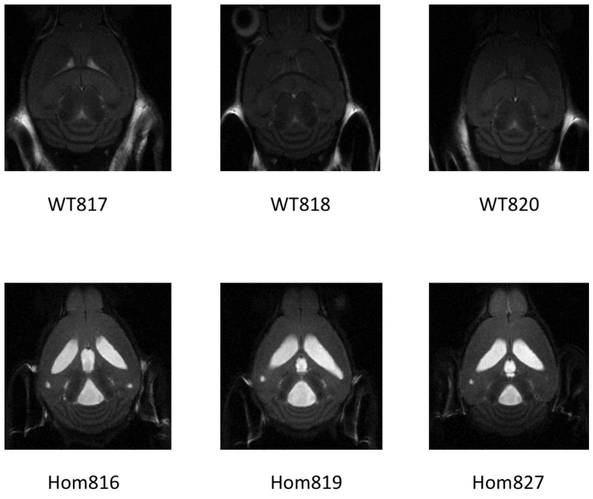

Figure. Brain MRI scans of 10-week old animals.

3) Although the authors clearly demonstrate that the nervous system is affected in this model, the analysis is a bit superficial. They also mention that the animals have defects in grip strength but show no data. The authors mention that a more thorough characterization of the CNS disease is underway. Hopefully, that study will include detailed regional (CNS and PNS) histomorphology, biochemical analyses, and a thorough behavioral/functional characterization.

Response: We are currently working on in-depth analyses of the central and peripheral nervous systems in this novel *Asah1*$^{P361R/P361R}$ mouse model. Compelling differences have been found so far in collaboration with Drs. James Eubanks at UHN and Steven Walkley at Einstein. Another manuscript will follow describing the behavioral and neural phenotypes, respectively, of this model.

<u>Minor comments:</u>

1) Were the mice transcardially perfused prior to collecting the various tissues for cytokine analysis? If so, the authors should mention this in the Materials and Methods. If not, this becomes problematic due to the increased cytokine levels in the serum.

Response: We repeated the MCP-1 measurements in spleen (WT, n=3; Homozygotes, n=4) and thymus lysates (WT and Homozygotes, n=3) collected after transcardially perfusing a new cohort of

© EMBO

animals with PBS. We still observed higher MCP-1 levels in the homozygous Asah1$^{P361R/P361R}$ mice animals compared to the WT (see Following Figure). We added these results to the text.

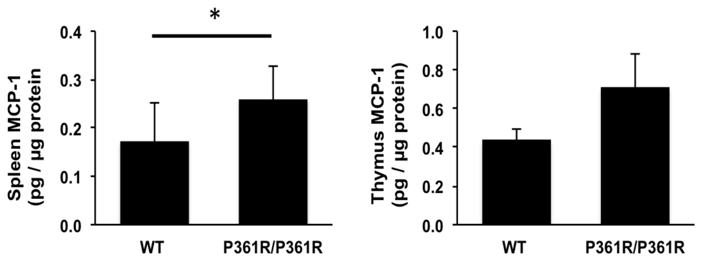

Figure. MCP-1 levels in spleens and thymus lysates from transcardially perfused animals.
Bars represent mean values. Error bars represent standard errors of the mean. **P < 0.05* for groups indicated.

2) Figure 4 refers to two methods, A and B, but only shows one data set.

Response: We thank the Reviewer for catching this omission. We have revised the Figure Legend accordingly.

3) Similarly, Figure 7 shows tissue ceramide levels for untreated and treated WT and Farber mice. Apparently, panels E and F show data from two independent methods. Showing both data sets is not necessary since the data appear to be virtually identical. The larger separate graphs (7E) demonstrate the data in a more convincing and understandable way. In addition, the text on pg 11 refers to the histology in Figure 7F. This will be correct if the authors delete the current Figure 7F (single graph, ceramide levels).

Response: We showed ceramide measurements by two independent methods to support our findings. According to the Reviewer's comments, we have now kept Panel E in Figure 7 and moved Panel F to the Supplementary Materials. Panel number ordering was then revised appropriately.

4) The arrows in Figures 5 and 7G are too small to see.

Response: We thank the Reviewer for these comments to improve the submission. The Figures were revised accordingly.

5) Although the authors show the life span curves for various groups of untreated and treated mice, they should formally state the exact median life spans in the text or Figure legends.

Response: The median survival was added to the text.

Comments to Reviewer 3

The use of standard error of the mean with an N=2 is not appropriate. Clarifying between which groups significance has been achieved is important and measuring not only if treated are different from untreated but also how close treated are to normal would be helpful.

Response: Please see Responses to these comments in the specific sections below.

© EMBO

1) Symptoms are subjective. Only humans can describe symptoms. Animals have clinical signs.

Response: The use of the word "symptoms", when describing animals, was revised in the manuscript and replaced with a more precise definition.

2) What is general dystrophy? What is pelvic prolapse and does it occur in patients? In males?

Response: "Dystrophy" refers to wasting of body tissues or organs. The *Asah1*$^{P361R/P361R}$ homozygous mice appear to have a generalized wasting of the body. Male *Asah1*$^{P361R/P361R}$ animals develop prolapse of the penis; we have added this precise definition to the manuscript. To the best of our knowledge, this condition has not been reported in patients.

3) It is not clear why the ovaries were examined so closely? "Furthermore, analyses of the ovaries from Asah1P361R/P361R animals support the previous finding that ACDase knockout leads to impairment of follicular development and therefore reduced fertility (Eliyahu et al., 2012)." But it is stated that KO mice did not survive beyond 4 cells.

Response: The knockout mice that did not survive beyond 4 cells were a result of a universal knockout of murine ACDase published by the lab of one of our co-authors. This finding was reported by Eliyahu et al. 2007 FASEB J. 21(7):1403-9. In a more recent study (Eliyahu et al., 2012 Cell Physiol Biochem. 30(3):735-48), the authors reported that ACDase is important in the development of ovaries. Conditional knockout of ACDase in the ovaries in that model led to reduced fertility. We were interested to see whether systemic accumulation of ceramide in our *Asah1*$^{P361R/P361R}$ mouse model would result in similar effects on the ovaries. We have revised the manuscript to make the distinction between the two studies clearer.

4) Mice cannot be "covered in fat". Please describe what is seen. Are alterations in fat distribution seen in human patients?

Response: We have revised this sentence as per the Reviewer's instruction. To the best of our knowledge, there are no reports describing fat distribution in Farber patients.

5) Hydrocephalus was seen in only one of two mice so the legend for figure 1: "Hydrocephaly was detected in the Asah1P361R/P361R mouse" needs to be more precise - Hydrocephaly was detected in an Asah1P361R/P361R mouse. Is hydrocephalus seen in human FB patients?

Response: We have imaged more animals to screen for hydrocephaly. The legend was modified accordingly (please see comments above for Referee #2). This condition was reported in one patient with Farber disease (Salo et al., 2003 J Pediatr Gastroenterol Nutr 36(2):274-7).

6) Page 6 - RBCs and hemoglobin were not only "slightly increased" but were significantly (P=<0.05) increased based on Figure 3B.

Response: The sentence in the text has been revised.

7) Where was the ceramide in the heart if "Rare scattered vacuolation of cardiac myofibers were interpreted to be within normal limits" but "total ceramide levels were dramatically elevated"?

Response: Ceramide levels were elevated in all organs examined including the heart. Examination of H&E-stained tissues showed that organs that were not infiltrated with macrophages sustained minimal to no structural damage. These organs included the heart, kidneys, and skeletal muscles. It is likely that macrophage infiltration of tissues is responsible for the damage seen in organs from the

homozygous *Asah1*$^{P361R/P361R}$ animals. Near-future studies by our laboratories will investigate the precise localization of ceramide storage in tissues using antibodies against ceramide.

8) Is there a toxic concern with lentivirus therapy as "Two Asah1P361R/P361R treated animals, one heterozygote, and one WT animal in the LV/ACDase-treated group demonstrated seizures at 3 weeks and were excluded from the growth curve analysis."? This should be discussed.

Response: No such toxicity has been reported from lentivector treatment and these gene delivery agents are now being used in the clinic for the treatment of LSDs. The animals that exhibited temporary seizures were from a big litter (15 pups). It is more likely that the seizures observed were due to early deprivation of nutritional factors as the animals recovered after weaning.

9) Describe more specifically the characteristics that defined the treated mice as "generally appeared healthier".

Response: More precise definitions have been added to the text.

10) While an increase in lifespan (when not euthanized at 10 weeks for pelvic prolapse) to 16.5 weeks is significant, it is still only a 50% increase in lifespan with therapy. Would this mean that children who die at two years might be expected to live until three? Is it know why they (children or mice) die?

Response: Our LV treatment was a one-time delivery of the therapeutic transgene as 'proof-of-principle' and not an optimized gene therapy or ERT treatment schema. Those in-depth studies are now underway in this novel *Asah1*$^{P361R/P361R}$ mouse model.

Farber disease is a systemic disorder where various organs are damaged. It is likely that death results from multiple factors rather than a single cause. We have not yet identified the exact cause of death in the homozygous *Asah1*$^{P361R/P361R}$ animals.

11) For the various graphs, it appears the statistics that are significant are between RV/en-treated and RV/ACDase-treated mice (as the P-value lines connect those two groups). Shouldn't differences or similarities be evaluated between treated and WT to determine how close (or not) the improvements have been towards normal?

Response: To avoid complexity in the analyses, we evaluated the improvements seen in the homozygous *Asah1*$^{P361R/P361R}$ animals treated with LV/ACDase in comparison to the homozygous *Asah1*$^{P361R/P361R}$ animals treated with LV/enGFP. This comparison diminishes variances due to contribution of the vector itself – especially as the intent here was 'proof-of-principle' that overexpression of the wild-type form of this transgene product affects outcome and (as was mentioned above) was not an optimized therapeutic treatment schema. Those studies (and such detailed comparisons) are currently underway in our laboratories. It is also mentioned in the text that treated mice manifested intermediate phenotypes. This applies to the different parameters we analyzed including: weight, survival, most peripheral blood differential counts, ceramide levels, and macrophage infiltrations.

12) It is not clear which groups are being evaluated in the body weight of Fig 7B. It would appear there are no differences between the LV/ACDase- and LV/enGFP-treated affected mice beyond 5 weeks (as stated in the text) but there are P=<0.001 indications for 9 and 10 weeks - that looks like it is between WT and treated.

Response: It is mentioned in the text that there are statistical differences in the body weights after 5

weeks between the two homozygous groups treated with either LV/enGFP or LV/ACDase. We revised the Figure to further clarify which groups are being evaluated.

13) Is the standard error of the mean really statistically informative with an N=2 (Figure 4)?

Response: The use of the standard error of the mean in an N=2 situation is statistically acceptable. The ceramide levels in all the tested organs from the homozygous mice were dramatically elevated. We use the standard error of the mean to show how close the results obtained from the two samples.

14) Figure 7D: Why do the authors postulate that basophils and esosinophils go up with LV/ACDase treatment in WT mice? Are lymphocytes now significantly lower than normal in LV-treated mice? They look lower in Fig. 7D but no statistical significance is indicated.

Response: We indeed observed significant elevations in basophils and eosinophils in WT animals treated with LV/ACDase compared to WT animals treated with LV/enGFP. We reported this finding in the manuscript given that ACDase upregulation has been shown in a number of studies to be involved in cell survival and proliferation. For clarity and to keep the story focused, we reported the outcome of the treatment on the significant differences between the two homozygous groups treated with either LV/ACDase or LV/enGFP.

Lymphocyte counts were significantly lower in homozygous mice (both LV/enGFP and LV/ACDase treated groups) compared to their WT counterparts. It should be noted that the blood analysis we did in the gene augmentation experiment was at a late point compared to the blood work we did in the initial assessment of the mouse phenotype. We suspect that this reduction in lymphocytes might be a result of a failing hematopoietic system at this late stage. A thorough analysis of hematopoiesis in this novel model is now underway in our laboratories.

15) Figure 7E would be improved by having WT and affected values for those not treated with any LV because the scale in Figure 4 is nmol/mg and in Figure 7E is pmol/mg. It would be best if the scales were the same. Figure 7F uses nmol/mg.

Response: The scale in Figure 7E was changed to nmol/mg.

16) Figure 7G: the arrows are very small and hard to see especially in the two spleen pictures.

Response: The Figure was revised as suggested.

17) "Microscopic examinations demonstrated reduction in macrophage infiltrations in the liver and spleen (Fig. 7F)" is actually Fig. 7G.

Response: The ordering of the Figure panels was revised as suggested.

18) In which tissues is the vector integrating - either actually in these experiments or from previous experiments?

Response: We refer the Reviewer to the paper from our lab by Yoshimitsu et al. (2004 Proc Natl Acad Sci USA 101(48):16909-14), wherein lentivector distribution after such neo-natal administration was analyzed in detail. We also provide this reference in the text.

19) Discussion: "We have recently completed experiments in enzymatically-normal non-human primates (NHPs) involving autologous transplantation of LV/ACDase-transduced hematopoietic cells. There we observed supranormal levels of ACDase-specific activity in the bone marrow, peripheral blood mononuclear cells, spleen, and liver. Reductions in ceramide levels were also observed (Walia et al., 2011)." Does this mean that ceramide levels in normal NHPs were reduced to below normal?

Response: In the study mentioned (Walia et al., 2011 Hum Gene Ther 22(6):679-87), ceramide levels in animals treated with LV/ACDase were indeed reduced in comparison to the levels observed in the control group that was treated with lentivectors engineering the expression of enGFP.

20) As you postulate that the failure to completely reverse the phenotype may be due to the human cDNA, are experiments planned or underway to use the mouse cDNA?

Response: We used the human cDNA in these studies as this orthologue of ACDase is more clinically relevant (for future human therapy trials; a major focus of our lab). That said, we have begun to engineer LVs that direct expression of the murine orthologue of ACDase to see in the future if there might be any such differences *in vitro* or *in vivo*.

21) How confident are the authors that the tissue destruction ("...widespread tissue destruction by lipid-laden macrophages...") is caused by macrophages and that it is not simply that macrophages are present as a response to tissue destruction and are there to scavenge necrotic tissue, which is a normal macrophage function?

Response: The Reviewer is correct – we do not know for sure yet whether the macrophage infiltration actually causes the tissue destruction we have observed in the model or is a secondary outcome. The text has been modified to decrease emphasis on this point. Along these lines, however, we observed that structural damage takes place in tissues from homozygous *Asah1*$^{P361R/P361R}$ animals that were infiltrated with macrophages. Organs that did not show macrophage infiltrates - under the light microscope – appeared structurally intact. Furthermore, granulomas formed by macrophages are attributed to the manifestations seen in Farber patients. Studies on the contribution of macrophages in this model are underway by our laboratories; for instance, by investigating how a blockade of MCP-1 signalling could interfere with the pathological (and clinical) signs in the affected *Asah1*$^{P361R/P361R}$ mice.

2nd Editorial Decision 20 March 2013

Thank you for the submission of your revised manuscript to EMBO Molecular Medicine. We have now received the enclosed reports from the referees that were asked to re-assess it. As you will see the reviewers are now globally supportive and I am pleased to inform you that we will be able to accept your manuscript pending the following final technical amendments:

1) I have noticed that while most images are generally of good quality, the text in many figures is rather blocky/blurry and does not hold well to magnification. In addition, figures 1 and 2 are overall of poor quality. Please provide higher resolution versions, and check to make sure that text/line-art remains clear even when zooming in. You may find that saving the images as EPS or PDF will better preserve the text and line-art resolution. If this does not help, you may need to remake the figures in a quality vector graphics program like Illustrator or the free opensource, alternative Inkscape. Please try improving them.

2) The description of all reported data that includes statistical testing must state the name of the statistical test used to generate error bars and P values, the number (n) of independent experiments underlying each data point (not replicate measures of one sample), and the actual P value for each test (not merely 'significant' or 'P < 0.05'). Please make sure that this if fully complied with.

3) There is space at the end of each article to list relevant web links for further consultation by our readers ("For more information"). Could you identify some relevant ones and provide such information as well? Some examples are patient associations, relevant databases, OMIM/proteins/genes links, author's websites, etc... (this is not compulsory but might apply in your case)

Please submit your revised manuscript within two weeks. Needless to say, the sooner we receive it the sooner I will be able to formally accept your manuscript.

I look forward to reading a new revised version of your manuscript as soon as possible.

***** Reviewer's comments *****

Referee #1 (Comments on Novelty/Model System):

See previous review

Referee #1 (General Remarks):

Again, the authors should be congratulated on an excellent piece of work, and on the conscientious way in which they addressed all of the comments raised in the first round of reviews.

Referee #2 (General Remarks):

The authors have thoughtfully and comprehensively addressed the concerns of the reviewers

Referee #3 (General Remarks):

The authors have satisfactorily answered my previous concerns.

2nd Revision - authors' response 04 April 2013

We are resubmitting a revised copy of the manuscript after incorporating all the technical changes suggested in your last email. Specifically, the following changes were made to the manuscript:

1. We saved the figures in pdf format to preserve the high resolution upon magnification.
2. We added the exact p values on the figures. Changes to the text/legends were made accordingly. The statistical tests used are mentioned under “Statistics” in the Materials and Methods.
3. The number of independent experiments underlying each data point was added to the figure legends.
4. In the Materials and Methods: we changed the subheading “Assessment of ACDase activity in lysates from mouse embryonic fibroblasts (MEFs)” to “Assessment of ACDase activity in intact mouse embryonic fibroblasts (MEFs)” for better accuracy.
5. We added links to the manuscript for related information on Farber disease, ACDase, structure predictions, and the Medin Lab.
6. We replaced the reference (Kienstra et al., 2007) with an earlier review from the group (Sands & Barker, 1999) that described the technique of neonatal gene transfer vector injection via the temporal vein.
