# Supplementary material for: Systemic ceramide accumulation leads to severe and varied pathological consequences
Source: EMBO Mol Med. 2013 May 16;5(6):827–42. doi: 10.1002/emmm.201202301 (PMC3779446; doi:10.1002/emmm.201202301)
Supplement: Supplementary file 2 [file emmm0005-0827-SD2.pdf]

Supplementary Material

Systemic Ceramide Accumulation Leads to Severe and Varied Pathological Consequences

Table of Contents:

Supplementary Figure 1. Schematic representation of homologous recombination between *Asah1* and the targeting vector.

Supplementary Figure 2. Evaluation of ceramide/DAG ratios.

Supplementary Figure 3. Western blot analysis of ACDase expression in kidneys.

Supplementary Figure 4. Evaluation of ceramide levels in *Asah1*$^{P361R/P361R}$ mice after lentivector treatment.

Supplementary Figure 5. Predicted structural model of P361R murine ACDase.

Supplementary Figure 6. Southern blot screening of ES cell clones.

Supplementary Figure 7. Fluorescent in situ hybridization (FISH) analysis.

Supplementary Table 1. Mendelian ratios of generated mice.

Supplementary Figure 1

A

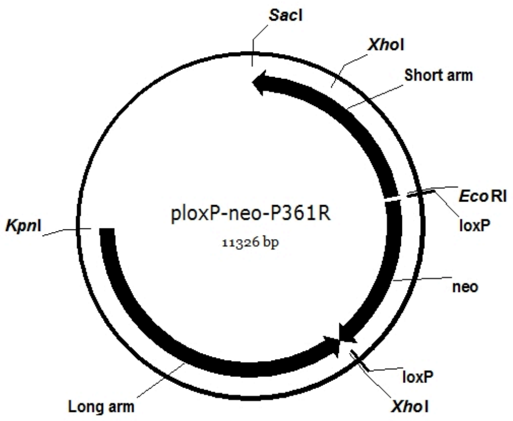

B

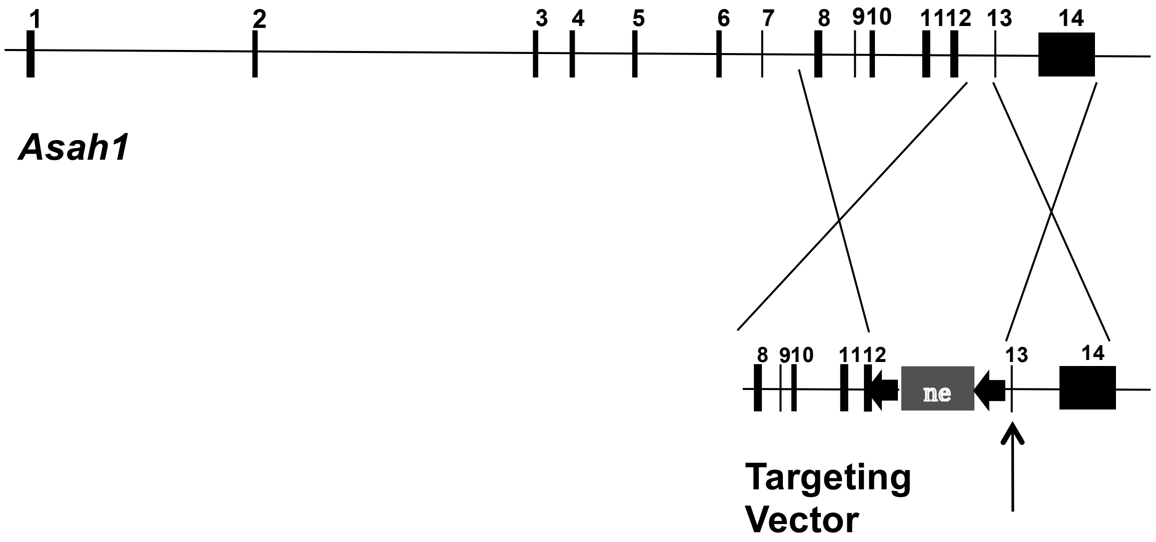

Supplementary Figure 1. Schematic representation of homologous recombination between *Asah1* and the targeting vector. (*A*) The non-linearized targeting vector plasmid consisting of a short arm (SA) and a long arm (LA) flanking a floxed neomycin-resistance cassette (neo^R). (*B*) Illustration of homologous recombination between the linearized targeting vector and *Asah1* gene. Arrow indicates the site of the mutation.

Supplementary Figure 2

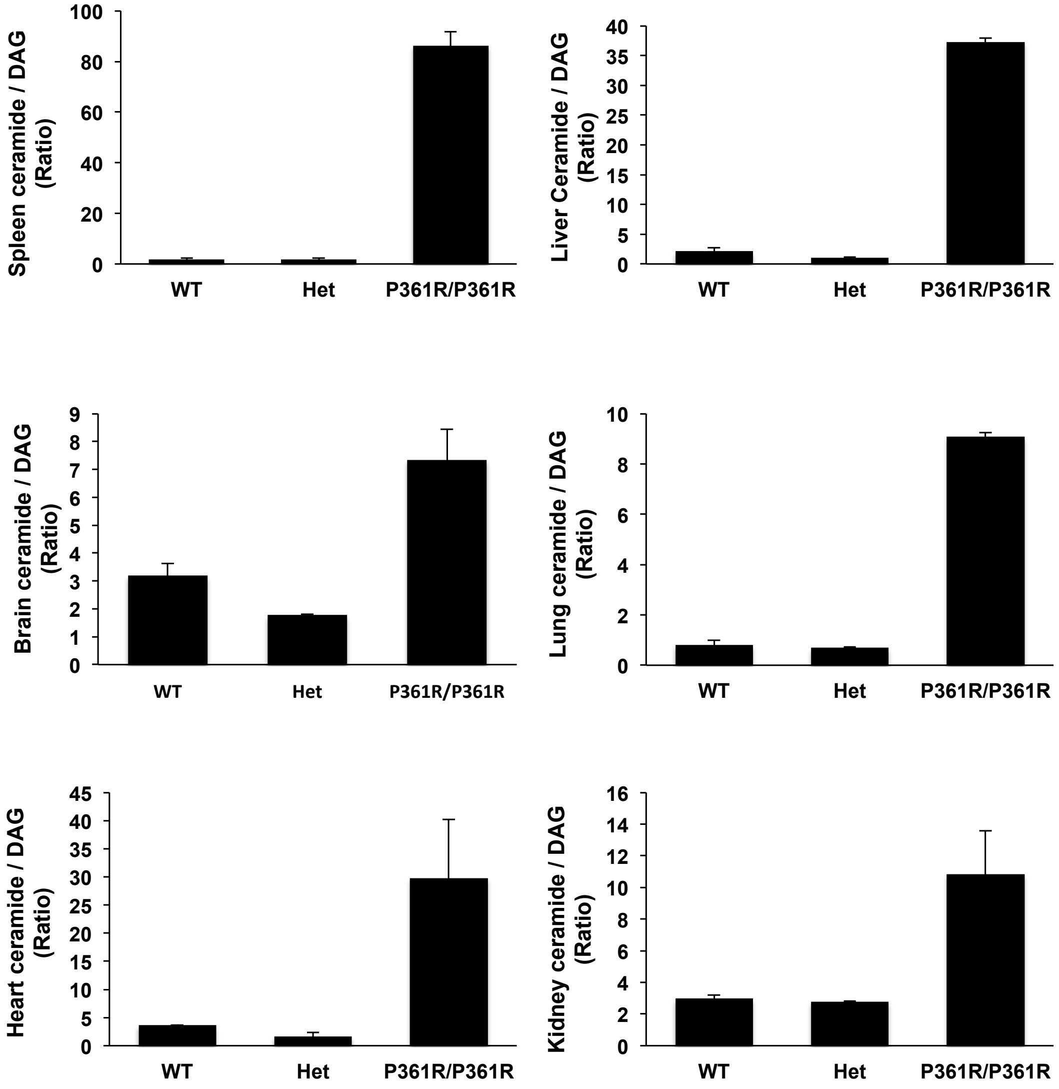

Supplementary Figure 2. Evaluation of ceramide/DAG ratios. Samples from spleens, livers, brains, lungs, hearts, and kidneys of 7-10 week-old mice were analyzed for ceramide-to-DAG ratios (n=2 for all genotypes). Bars represent mean values. Error bars represent standard errors of the mean.

Supplementary Figure 3

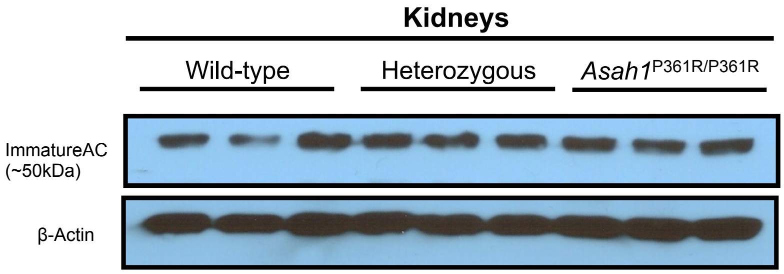

Supplementary Figure 3. Western blot analysis of ACDase expression in kidneys. Kidneys from WT, Het, and *Asah1*$^{P361R/P361R}$ mice were harvested at 7-9 weeks of age. The immature form of the mACDase protein was detected.

Supplementary Figure 4 (A)

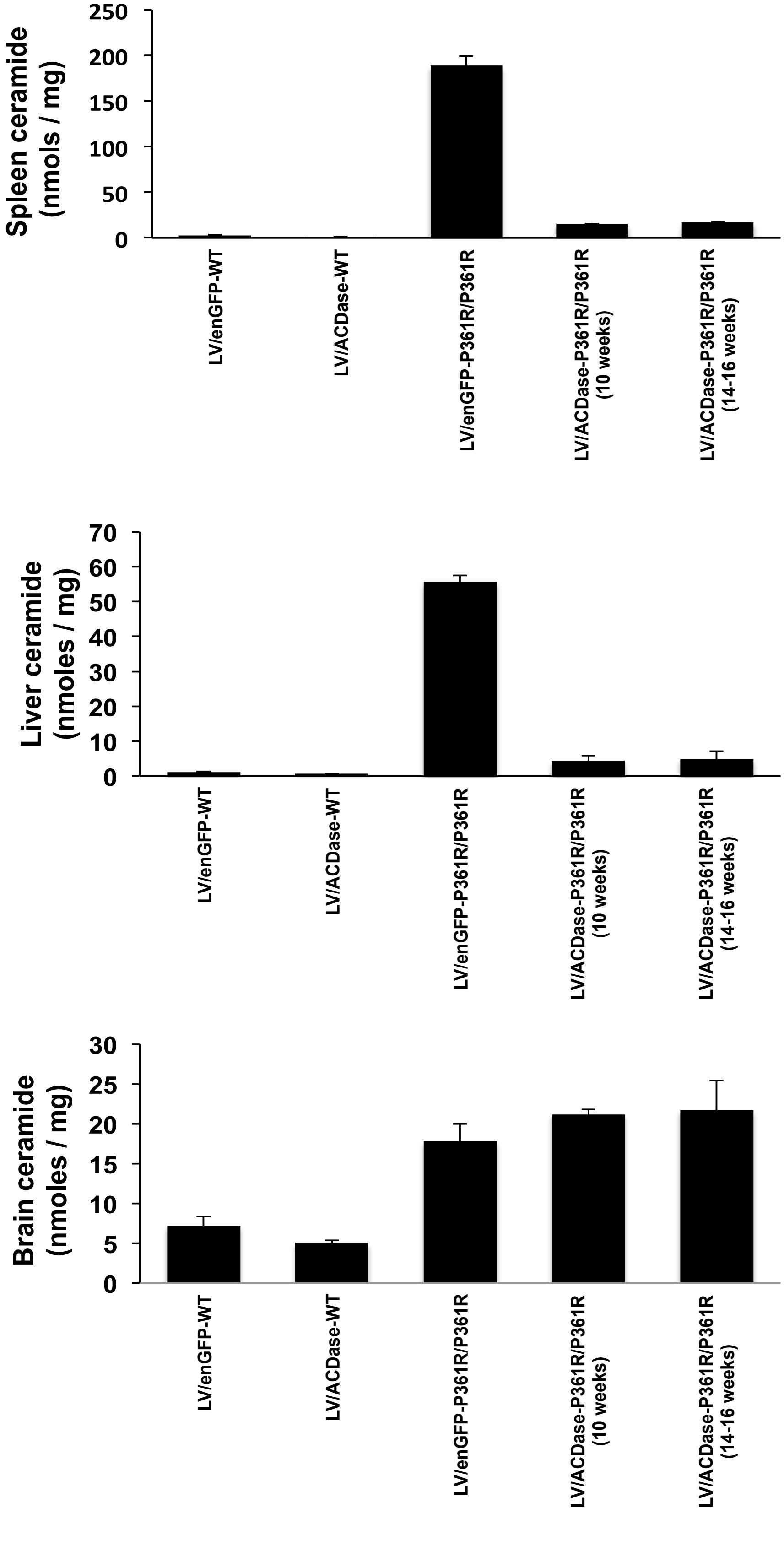

Supplementary Figure 4

B

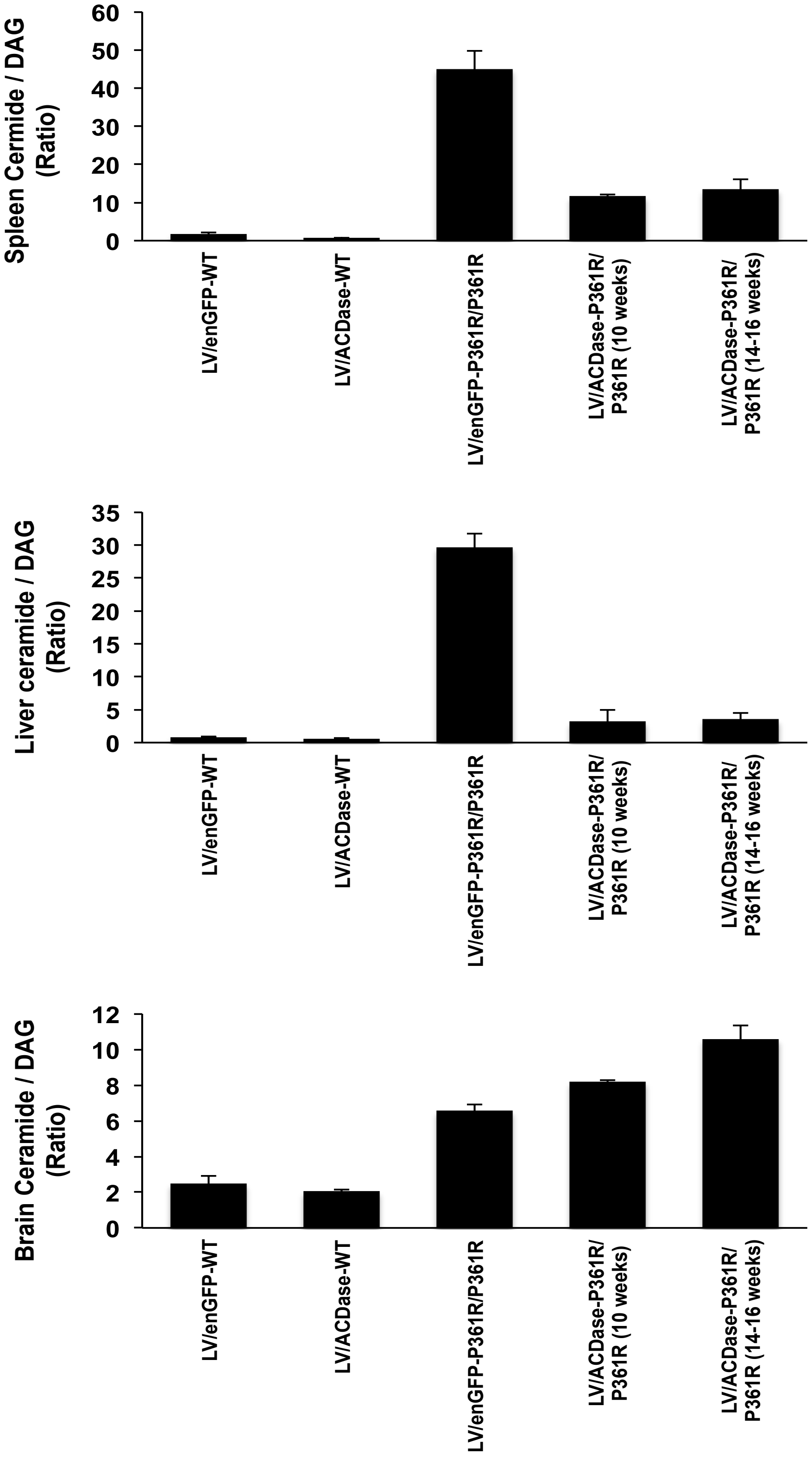

Supplementary Figure 4. Evaluation of ceramide levels in *Asah1*$^{P361R/P361R}$ mice after lentivector treatment. Samples from spleens, livers, and brains of 10 week-old LV/enGFP-WT, LV/ACDase-WT, LV/enGFP-*Asah1*$^{P361R/P361R}$, and LV/ACDase-*Asah1*$^{P361R/P361R}$ mice, and 14-16-week old LV/ACDase-*Asah1*$^{P361R/P361R}$ mice were analyzed for (*A*) total ceramide levels using *E.coli* DAG kinase assay and (*B*) ceramide/DAG ratios. N=2 for all groups. Bars represent mean values. Error bars represent standard errors of the mean.

Supplementary Figure 5

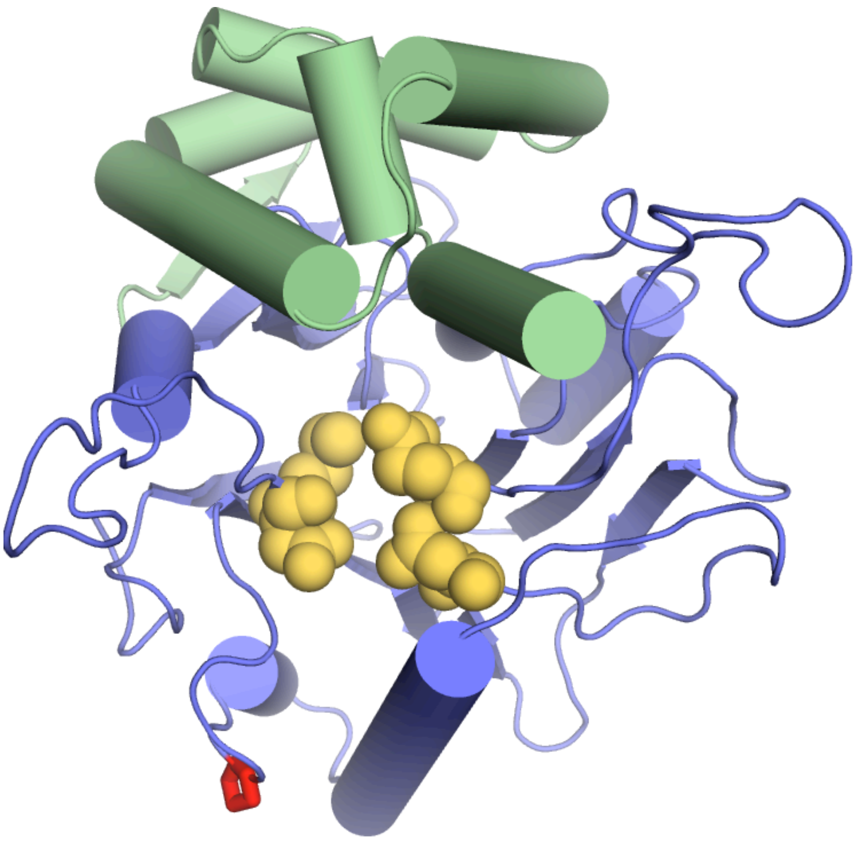

Supplementary Figure 5. Predicted structural model of P361R murine ACDase. The α subunit is shown in green and the β subunit is in blue. Putative active site residues are shown as yellow spheres. The mutated proline 361 residue is shown in red.

Supplementary Figure 6

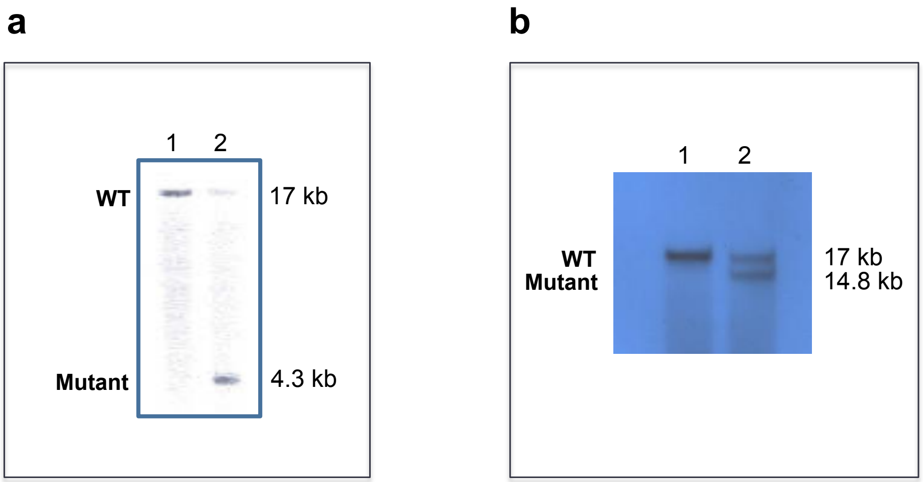

Supplementary Figure 6. Southern blot screening of ES cell clones. DNA from WT and *Asah1*$^{P361R/P361R}$ ES-cell clones were digested with the HindIII enzyme and examined by Southern blot using a 3' probe (*A*) and a 5' probe (*B*). The downstream probe detected 4.3- and 7-kb fragments representing the targeted and the WT alleles, respectively. The result was confirmed using the upstream probe, which detected 14.3- and 17-kb fragments representing the targeted and WT alleles, respectively. Sample (1) is a control showing only the WT allele and sample (2) is a variant clone showing both WT and mutant alleles indicating heterozygosity.

Supplementary Figure 7

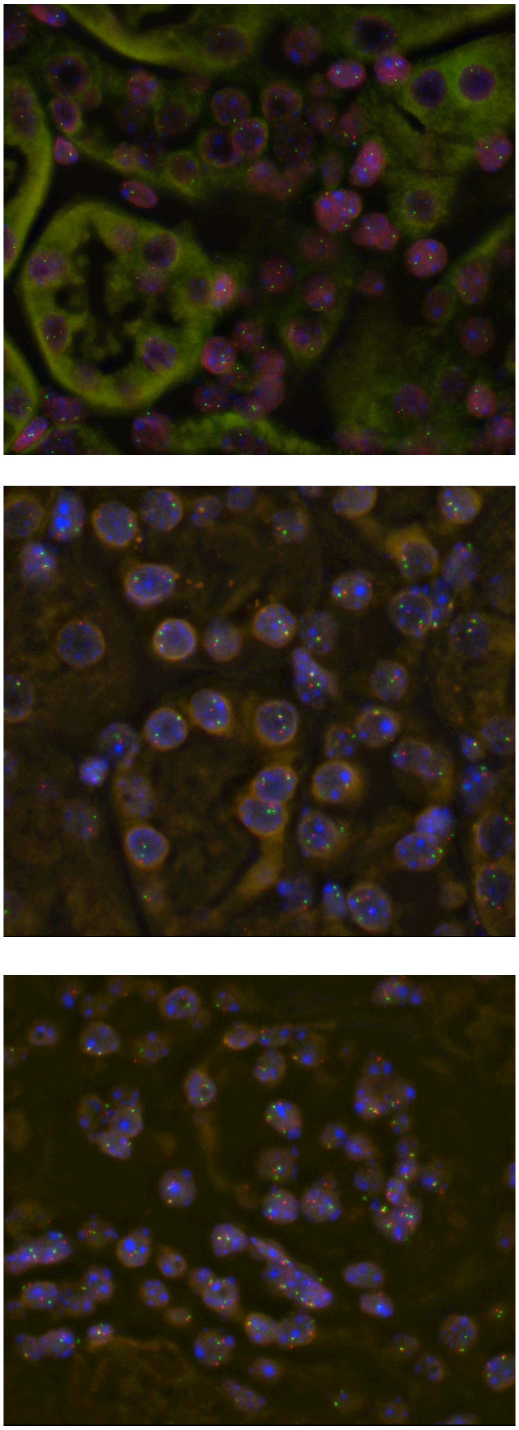

WT

Het

***Asah1*$^{P361R/P361R}$**

Supplementary Figure 7. Fluorescent in situ hybridization (FISH) analysis. Kidneys were harvested from 9-week old WT, Het, and *Asah1*$^{P361R/P361R}$ mice. Samples were then analyzed using FISH for gene copy number. Green indicates the *Lamp-1* gene and red represents the *Asah1* gene. No off-target *Asah1* sequences were observed.

Supplementary Table1

	Expected	Generated
WT	0.25	0.175
Het	0.50	0.554
P361R/P361R	0.25	0.268

Supplementary Table 1. Mendelian ratios of generated mice. Heterozygotes were intercrossed and ratios of WT, heterozygous, and *Asah1*$^{P361R/P361R}$ mice obtained were calculated (total n = 257).
